# LINC00460/miR-186-3p/MYC feedback loop facilitates colorectal cancer immune escape by enhancing CD47 and PD-L1 expressions

**DOI:** 10.1186/s13046-024-03145-1

**Published:** 2024-08-13

**Authors:** Qingqing Luo, Fei Shen, Sheng Zhao, Lan Dong, Jianchang Wei, He Hu, Qing Huang, Qiang Wang, Ping Yang, Wenlong Liang, Wanglin Li, Feng He, Jie Cao

**Affiliations:** 1 Department of Gastroenterology and Hepatology, Guangzhou First People’s Hospital, School of Medicine, South China University of Technology, Guangzhou, Guangdong 510180 China; 2 Guangzhou Key Laboratory of Digestive Diseases, Guangzhou Digestive Disease Center, Guangzhou First People’s Hospital, School of Medicine, South China University of Technology, Guangzhou, Guangdong 510180 China; 3 Guangzhou Digestive Disease Center, Department of Gastrointestinal Surgery, The Second Affiliated Hospital, School of Medicine, South China University of Technology, Guangzhou, Guangdong 510180 China; 4 Department of General Surgery, The First Affiliated Hospital, Jinan University, Guangzhou, Guangdong 510630 China; 5 Department of Thyroid surgery, the Second Affiliated Hospital, School of Medicine, South China University of Technology, Guangzhou, Guangdong 510180 China; 6 Department of Nephrology, The Second Affiliated Hospital, School of Medicine, South China University of Technology, Guangzhou, Guangdong 510180 China

**Keywords:** Colorectal cancer, LINC00460, miR-186-3p, MYC, CD47, PD-L1

## Abstract

**Background:**

Long non-coding RNAs (LncRNAs) have been implicated as critical regulators of cancer tumorigenesis and progression. However, their functions and molecular mechanisms in colorectal cancer (CRC) still remain to be further elucidated.

**Methods:**

LINC00460 was identified by differential analysis between human CRC and normal tissues and verified by in situ hybridization (ISH) and qRT-PCR. We investigated the biological functions of LINC00460 in CRC by in vitro and in vivo experiments. We predicted the mechanism and downstream functional molecules of LINC00460 by bioinformatics analysis, and confirmed them by dual luciferase reporter gene assay, RNA immunoprecipitation (RIP), RNA pull-down, etc.

**Results:**

LINC00460 was found to be significantly overexpressed in CRC and associated with poor prognosis. Overexpression of LINC00460 promoted CRC cell immune escape and remodeled a suppressive tumor immune microenvironment, thereby promoting CRC proliferation and metastasis. Mechanistic studies showed that LINC00460 served as a molecular sponge for miR-186-3p, and then promoted the expressions of MYC, CD47 and PD-L1 to facilitate CRC cell immune escape. We also demonstrated that MYC upregulated LINC00460 expression at the transcriptional level and formed a positive feedback loop.

**Conclusions:**

The LINC00460/miR-186-3p/MYC feedback loop promotes CRC cell immune escape and subsequently facilitates CRC proliferation and metastasis. Our findings provide novel insight into LINC00460 as a CRC immune regulator, and provide a potential therapeutic target for CRC patients.

**Supplementary Information:**

The online version contains supplementary material available at 10.1186/s13046-024-03145-1.

## Introduction

Colorectal cancer (CRC) is one of the most lethal malignancies worldwide, and the leading cause of cancer death in China [1–3]. The incidence and mortality of CRC rank third and second, respectively, among all malignant tumors in the world [4]. The difficulty of early detection and the tendency of late recurrence and metastasis are the main reasons for the poor prognosis of CRC patients [5–7]. Therefore, it’s still a big challenge to identify the early diagnostic biomarkers and later effective therapeutic targets for CRC patients.

Long non-coding RNAs (LncRNAs) are a special class of transcripts with a length of more than 200 nucleotides [8, 9]. LncRNAs generally lack of protein-coding ability and function in epigenetic, transcriptional and post-transcriptional regulation [10–16]. There is increasing evidence that LncRNAs play an important role in malignant tumors [17, 18]. For example, RP11 [19, 20], H19 [21, 22], RAMS11 [23], PVT1 [24, 25] have been reported as tumor promoters, and MIR22HG [26], GAS5 [27] as suppressors in CRC.

Furthermore, since 2017, immunotherapy has been beneficial for some patients with advanced cancers, including CRC [28]. However, immunotherapy only works for 15% of dMMR-MSI-H CRC patients, and 85% of pMMR-MSI-L patients were generally resistant to immunotherapy [29, 30]. Therefore, it is crucial to further explore more novel immune-related biomarkers and therapeutic targets to improve immunotherapy response rates. Recently, researchers have shown that some LncRNAs may have an influence on the immune system and immunotherapy [31–33]. Ni, W. et al. uncovered that LncRNA SNHG29-mediated YAP activation leads to suppressive tumor immunity by promoting PD-L1 expression [34]. Xu, M. et al. revealed that LncRNA SATB2-AS1 inhibits CRC cell metastasis and regulates TH1-type chemokine expression and immune cell density in CRC [35]. Lin, Z. et al. demonstrated that LncRNA KCNQ1OT1 mediates CD8^+^ T cell exhaustion by regulating CD155 expression in CRC [36].

Here, we discovered that LINC00460 was highly upregulated in CRC and correlated with a poor prognosis. This research demonstrated that LINC00460 promoted CRC cell immune escape by increasing the expressions of MYC, CD47 and PD-L1 in CRC cells and remodeling the CRC tumor-inhibitory immune microenvironment, thereby promoting the occurrence and development of CRC tumors. We also found that MYC, as a transcription factor, increased the expression level of LINC00460. In conclusion, our study demonstrated that LINC00460 served as a CRC immune regulator through the LINC00460/miR-186-3p/MYC feedback loop, providing a novel therapeutic strategy and target for CRC.

## Materials and methods

### Genome-wide expression of LncRNAs in CRC from public databases

We downloaded the genome-wide expression profiles across 469 colon adenocarcinoma (COAD) patients and 39 normal controls, and 163 rectum adenocarcinoma (READ) patients and 9 normal controls from the UCSC Xena project (GDC TCGA, http://xena.ucsc.edu/). And we searched nine datasets of 2595 samples from the Gene Expression Omnibus (GEO, https://www.ncbi.nlm.nih.gov/geo), among which GSE109454 was previously published by our team [24]. In addition, the corresponding clinical information (age, sex, TNM stage, survival status, survival time, etc.) of CRC patients was also downloaded for further analysis. Supplementary Table S1 summarizes the source and details of the included datasets and samples.

### Human sample tissues

Matched human CRC and normal colon or rectum tissues from 129 patients and their clinical information were collected from the Guangzhou First People’s Hospital from November 2018 to July 2021. Among these patients, there were 10 CRC patients with liver metastases and another 10 patients with lung metastases. The fresh surgical specimens were brought to the laboratory by storing in liquid nitrogen. Each tissue was divided into two pieces, one was used to prepare a formalin-fixed, paraffin-embedded (FFPE) sample for in situ hybridization and pathological staining, and the other was quickly stored in -80 °C refrigerator for total RNA or protein extraction. These tissues were not be repeatedly frozen and thawed to truly reflect in vivo RNA levels. Our study was conducted with the approval of the Ethics Committee of Guangzhou First People’s Hospital and with the informed consent of the patients.

### Cell culture

NCM460 was purchased from EK-Bioscience (CC-Y1550, Shanghai, China). And HCT116, HT29, SW480 and SW620 were purchased from iCell Bioscience (iCell-h071, -h078, -h204 and -h206, Shanghai, China). MC38 was purchased from zqxzbio (ZQ0933, Shanghai, China). All cells were cultured in DMEM/high glucose (Gibco, New York, USA) with 10% fetal bovine serum (Biological Industries, Cromwell, USA) at 5% CO2 saturation humidity at 37 °C.

### Animal experiments

Four-week-old female BALB/c nude mice were purchased from Slack (Guangzhou, China), and eight-week-old male C57BL/6J mice were derived from GuangDong GemPharmatech Co., Ltd. All mice were housed in an SPF barrier under standard conditions. Groups of MC38-Luc-LINC00460, MC38-Luc-vector, MC38-Luc-sh-LINC00460 and MC38-Luc-sh-NC cells (1 × 10^6^) were injected subcutaneously into the right flank of 4-week-old female BALB/c nude mice. Tumor volumes were monitored every 3 days and calculated as 0.5 × length × width^2^. After 28 days, mice were sacrificed and tumors were harvested for further analysis.

Groups of MC38-Luc-LINC00460 and MC38-Luc-vector cells (1 × 10^6^) were injected into the inferior mesenteric vein of 8-week-old male C57BL/6J mice to establish liver metastases. Groups of MC38-Luc-sh-LINC00460 and MC38-Luc-sh-NC cells (1 × 10^6^) were injected into the tail vein of 8-week-old male C57BL/6J mice to establish lung metastases. Bioluminescence images were acquired using PerkinElmer IVIS Spectrum system. Mice were sacrificed and liver or lung metastases were excised after 20 or 16 days, respectively. The animal experiments were approved by the Animal Ethics Committee of the South China University of Technology, and the principles of animal welfare were followed.

### Statistical analysis

All data processing, statistical analysis, and visualization were performed using GraphPad Prism 7.0 and R studio 4.0.0 software. Each result was obtained from at least three independent replicate experiments. Student’s t test and one-way analysis of variance (ANOVA) were used to compare two or more groups of continuous variables, respectively. Chi-squared test was applied to compare categorical variables for statistical analysis. Survival curves were plotted using the Kaplan-Meier method and compared with log-rank test. Correlation analyses between two continuous variables were assessed via Pearson’s correlation coefficients. All statistical tests were two- tailed, and *P* < 0.05 was considered statistically significant. Data are presented as mean ± standard deviation (SD), ns *p* ≥ 0.05, * *p* < 0.05, ** *p* < 0.01, *** *p* < 0.001, **** *p* < 0.0001.

### Supplementary materials and methods

For details on nuclear and cytoplasmic RNA extraction, vector construction and cell transfection, in situ hybridization (ISH) and immunohistochemistry (IHC), total RNA isolation and quantitative real-time PCR (qRT-PCR), cell proliferation, migration and invasion assays, Western blot (WB), luciferase activity assays, RNA immunoprecipitation (RIP), RNA pull-down assay, please see Supplementary Materials and Methods.

## Results

### Integrative analysis reveals LINC00460 as a tumor promoter in CRC

**Fig. 1:**
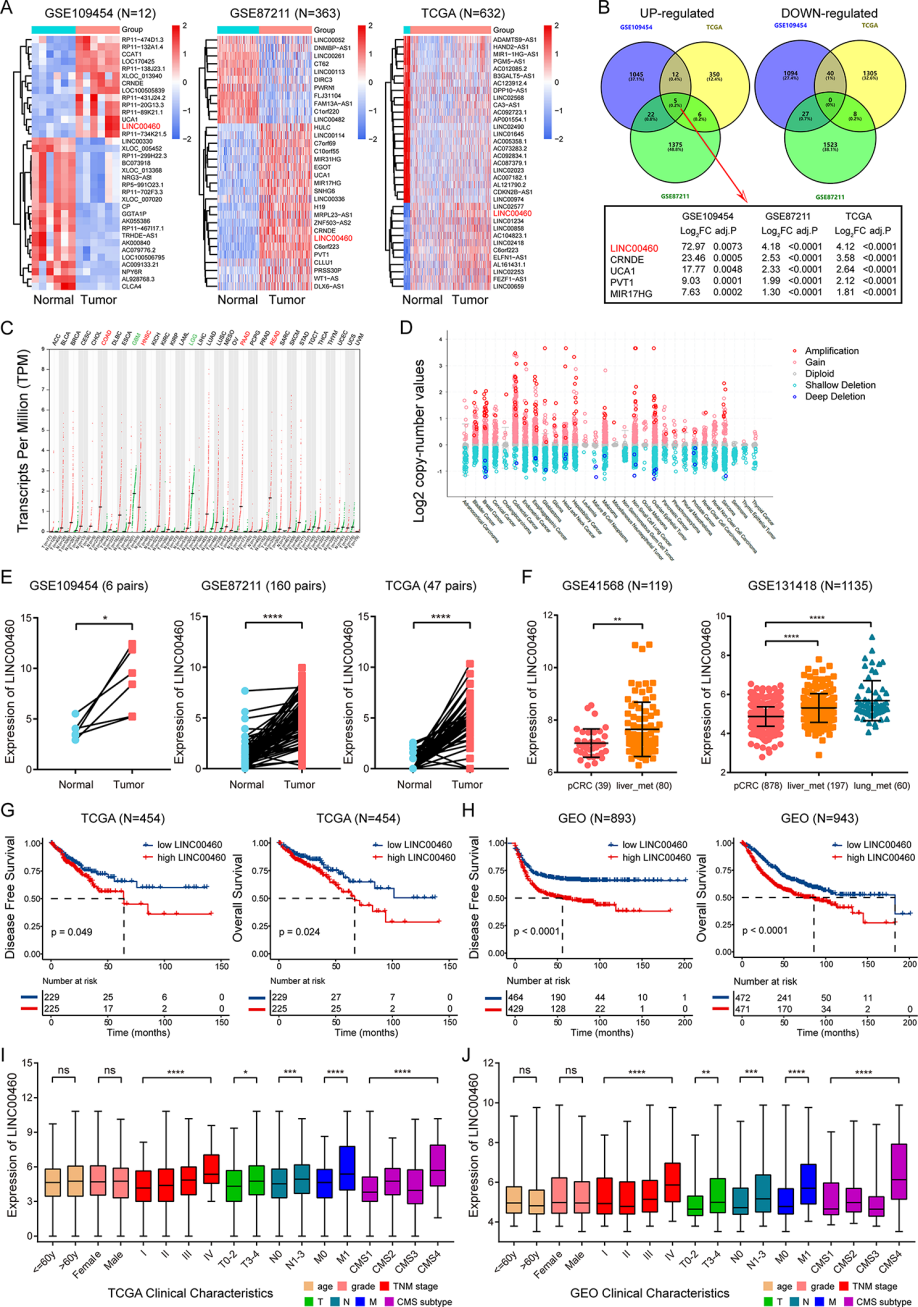
LINC00460 is overexpressed in CRC and associated with poor prognosis. **(A)** Hierarchical clustering heatmaps of differentially expressed LncRNAs between CRC tissues and normal colon controls in GSE109454 (*N* = 12), GSE87211 (*N* = 363) and TCGA database (*N* = 632). (**B)** Venn diagram showed the intersection of up-regulated and down-regulated LncRNAs in the three datasets. **(C)** The expression of LINC00460 in 33 kinds of tumors compared with normal tissues from GEPIA2 website. **(D)** The copy number variation of LINC00460 in 30 tumors from cBioPortal website. **(E)** The expression levels of LINC00460 in paired CRC tumors and normal controls in GSE109454 (6 pairs), GSE87211 (160 pairs) and TCGA database (47 pairs). **(F)** The expression levels of LINC00460 in primary CRC, liver and lung metastases in GSE41568 (*N* = 119, pCRC = 39, liver_met = 80) and GSE131418 (*N* = 1135, pCRC = 878, liver_met = 197, Lung_met = 60). **(G**,** H)** Kaplan-Meier analysis of the correlation between LINC00460 levels and disease-free survival or overall survival in TCGA (*N* = 454) and GEO databases (*N* = 893/943). Log-rank test was used to estimate the significance. **(I**,** J)** The expression levels of LINC00460 classified by different clinicopathological features in TCGA and GEO databases. Data were presented as mean ± standard deviation (SD), ns *p* ≥ 0.05, *p < 0.05, **p < 0.01, ***p < 0.001, ****p < 0.0001

Given the critical role of LncRNAs in tumor pathogenesis [17, 37, 38], we aimed to identify LncRNAs that potentially drive colorectal tumor initiation and progression. To screen for overexpressed LncRNAs in CRC, microarray analysis was applied to compare LncRNA expression profiles in CRC and normal controls. Hierarchical clustering revealed differences in LncRNA expression between the two groups in GSE109454, GSE87211 and TCGA datasets with a cut-off criterion of fold change > 2.0 and *p* < 0.05 (Fig. 1A). The intersection of Venn plots showed that five LncRNAs were simultaneously up-regulated in CRC of the three datasets, among which LINC00460 had the highest fold change (Fig. 1B). We then queried GEPIA2 (http://gepia2.cancer-pku.cn/#index) and found that LINC00460 was significantly highly expressed in both COAD and READ (Fig. 1C, Fig. S1). Furthermore, investigation of LINC00460 copy number variation (CNV) across 30 cancer types in cBioPortal (https://www.cbioportal.org/) showed a prominent copy number amplification in colorectal cancer (Fig. 1D). LINC00460 was found to be significantly overexpressed in CRC compared to matched normal tissues in GSE109454, GSE87211 and TCGA (Fig. 1E). LINC00460 was also sharply upregulated in CRC liver and lung metastases compared to primary lesions in GSE41568 and GSE131418 (Fig. 1F).

Notably, Kaplan-Meier survival curves and log-rank tests revealed that high LINC00460 expression was significantly positively associated with decreased the disease-free survival (DFS) and overall survival (OS) of CRC patients in both TCGA and GEO databases (Fig. 1G-H). Moreover, we investigated the association between LINC00460 and clinicopathological characteristics of CRC patients in both TCGA and GEO databases. The results showed that high LINC00460 expression was significantly correlated with poor clinical stage or molecular subtype, suggesting that high LINC00460 expression may predict a worse prognosis of CRC patients (Fig. 1I-J). Taken together, these observations indicate that LINC00460 may play a role as a tumor promoter and may serve as a novel prognostic marker in CRC.

### Validation of LINC00460 expression and cellular localization

**Fig. 2:**
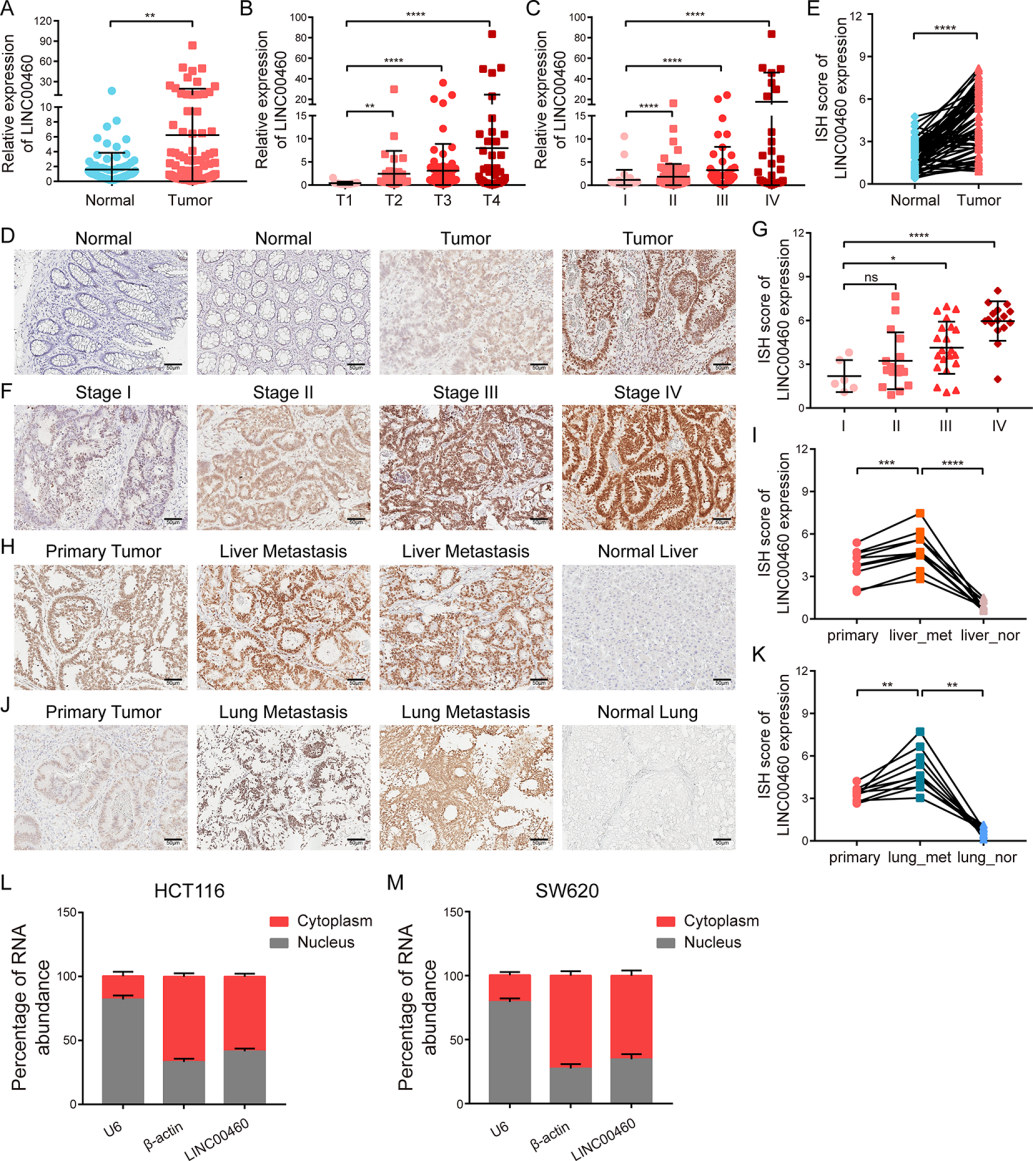
Validation of the LINC00460 upregulation in CRC and its cellular localization. **(A)** The expression of LINC00460 in 102 pairs of human CRC and normal tissues were detected by qRT-PCR. 18 S was used as an internal reference. **(B-C)** qRT-PCR analyses showed the LINC00460 expression level in different T stages (T1 = 17, T2 = 39, T3 = 73, T4 = 47) and TNM stages (I = 29, II = 71, III = 49, IV = 28). **(D**,** E**,** F**,** G**,** H**,** I**,** J**,** K)** ISH images and scores measured the expression of LINC00460 in matched human CRC tissues and normal controls (*N* = 63 pairs, **D**,** E**), human CRC tissues of different TNM stages (I = 6, II = 16, III = 21, IV = 16, **F**,** G**), matched primary CRC tumors, liver metastases, and normal liver tissues (*N* = 10 groups, **H**,** I**), and matched primary CRC tumors, lung metastases, and normal lung tissues (*N* = 10 groups, **J**,** K**). Magnification, 200 x, scale bar, 50 μm. **(L-M)** The expression levels of LINC00460 in the nucleus and cytoplasm were measured by qRT-PCR with U6 and β-actin as internal references in HCT116 and SW620 cells, respectively (*N* = 3). Data were presented as mean ± standard deviation (SD), ns *p* ≥ 0.05, *p < 0.05, **p < 0.01, ***p < 0.001, ****p < 0.0001

To further validate the differential expression of LINC00460, qRT-PCR was performed on 102 pairs of matched CRC and normal tissues, and the results showed that CRC tissues had a significantly higher expression level of LINC00460 (Fig. 2A). Intriguingly, the LINC00460 expression of the 102 CRC tissues was significantly positively correlated with T and TNM stage (Fig. 2B-C). Furthermore, we performed ISH assays on paraffin-embedded tissue sections of normal colorectal controls, CRC tissues, liver or lung metastases and normal liver or lung tissues, respectively. ISH images and scores showed that CRC tissues had higher LINC00460 expression than normal colorectal tissues (Fig. 2D-E), and high LINC00460 expression was significantly associated with poor TNM stage (Fig. 2F-G). Simultaneously, liver or lung metastases enriched more LINC00460 than their matched primary lesions and normal liver or lung tissues, respectively (Fig. 2H-K).

To observe the cellular localization of LINC00460, qRT-PCR analysis was performed for nuclear and cytoplasmic LINC00460 in HCT116 and SW620 cells, and the results showed that LINC00460 was mainly located in the cytoplasm (Fig. 2L-M). Accordingly, LINC00460 was confirmed to be overexpressed in CRC primary lesions and metastases and was predominantly located in the cytoplasm.

### LINC00460 promotes CRC cell proliferation, migration and invasion in vitro

**Fig. 3:**
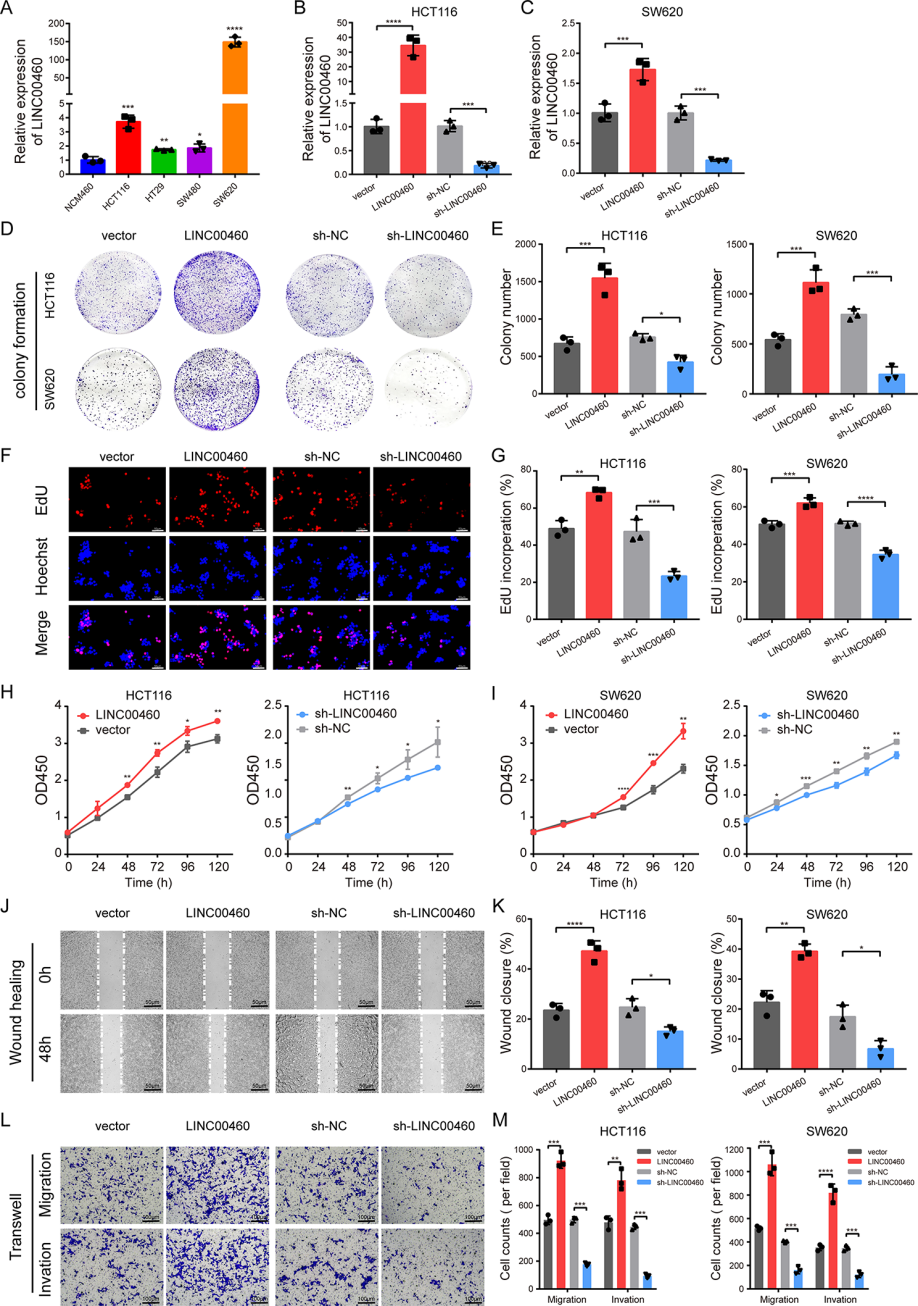
LINC00460 promotes CRC cell proliferation, migration and invasion in vitro. **(A)** The expression of LINC00460 in NCM460, HCT116, HT29, SW480 and SW620 cell lines was detected by qRT-PCR. 18 S was used as an internal reference. **(B-C)** qRT-PCR analysis revealed the expression of LINC00460 in HCT116 and SW480 cells stably transfected with LINC00460 overexpression or shRNA plasmid compared with their control vectors. **(D**,** E**,** F**,** G**,** H**,** I)** Colony formation, EdU fluorescence staining and CCK-8 assays detected the cell proliferation ability of transfected HCT116 and SW620 (*N* = 3, magnification, 200 x, scale bar, 50 μm). **(J**,** K**,** L**,** M)** Cell migration and invasion abilities were determined by wound healing and transwell assays in transfected HCT116 and SW620 cells (*N* = 3, magnification, 200 x, scale, 50 μm). Data were presented as mean ± standard deviation (SD), ns *p* ≥ 0.05, *p < 0.05, **p < 0.01, ***p < 0.001, ****p < 0.0001

To explore the biological functions of LINC00460 in vitro, we first measured the baseline levels of LINC00460 in four human CRC cell lines (HCT116, HT29, SW480, SW620) and one normal colon epithelial cell line (NCM460). The results of the qRT-PCR analyses showed that LINC00460 expression was markedly increased in four CRC cell lines compared to NCM460 (Fig. 3A). We then applied HCT116 and SW620 to construct cell lines with stable LINC00460 overexpression or knockdown via overexpression or shRNA lentivirus (Fig. 3B-C). Colony formation assays showed that the cell colony number of HCT116 and SW620 were sharply increased by up-regulation of LINC00460 and markedly impaired by depleted of LINC00460 (Fig. 3D-E). EdU fluorescence staining revealed that overexpression or knockdown of LINC00460 significantly increased or decreased the proportion of EdU-positive cells (Fig. 3F-G). Similarly, growth curves of CCK-8 assays showed that LINC00460 up- or down-regulation significantly enhanced or inhibited the proliferation capacity of HCT116 and SW620 cells (Fig. 3H-I).

Furthermore, the effects of LINC00460 on migration and invasion of CRC cells were also evaluated. The wound healing assays and transwell analyses revealed that LINC00460 overexpression triggered the migration and invasion abilities of both HCT116 and SW620 cells, and LINC00460 knockdown inhibited the in vitro migration and invasion (Fig. 3J-M). Taken together, these results indicate that LINC00460 promotes CRC tumorigenesis and metastasis in vitro.

### LINC00460 promotes CRC tumorigenesis, metastasis, and angiogenesis in vivo

**Fig. 4:**
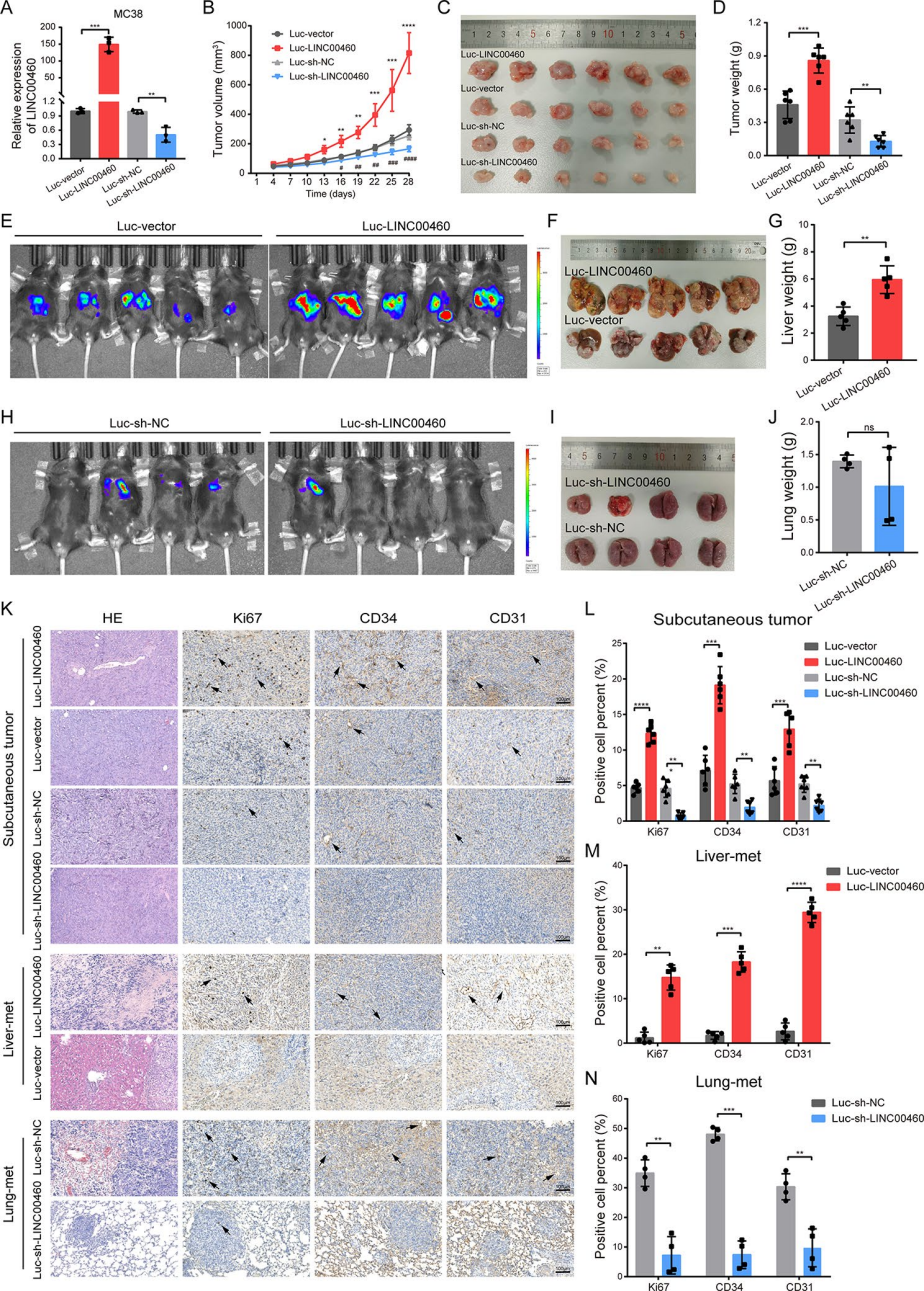
LINC00460 promotes tumorigenesis, metastasis and angiogenesis of CRC in vivo. **(A)** qRT-PCR analysis measured the expression level of LINC00460 in MC38 cells stably transfected with luciferase and LINC00460 overexpression or shRNA plasmid compared with their control vectors (*N* = 3). **(B)** The growth curves of subcutaneous tumors measured every 3 days (*N* = 6). **(C-D)** Images and body weight of subcutaneous tumors of each group (*N* = 6). **(E**,** H)** Bioluminescent images of livers (*N* = 5, **E**) or lungs (*N* = 4, **H**) for each experimental group on 20th or 16th day. **(F-G**,** I-J)** Images and organ weight of liver (*N* = 5, **F-G**) or lung (*N* = 4, **I-J**) metastases of each experimental group. **(K**,** L**,** M**,** N)** H&E staining and IHC assays of Ki67, CD34 and CD31 evaluated the metastatic nodules, cell proliferation and angiogenesis in subcutaneous tumors, liver or lung metastases of each experimental group. Magnification, × 100, scale, 100 μm. Data were presented as mean ± standard deviation (SD), ns *p* ≥ 0.05, *p < 0.05, **p < 0.01, ***p < 0.001, ****p < 0.0001

To determine the effect of LINC00460 on tumor growth, metastasis and angiogenesis in vivo, we constructed MC38 cells stably transfected with Luc-vector, Luc-LINC00460, Luc-sh-NC and Luc-sh-LINC00460, respectively (Fig. 4A). The four groups of MC38 cells were each injected subcutaneously into 4-week-old female nude mice, respectively. The growth curves and tumor volume and weight showed that overexpression of LINC00460 significantly promoted the subcutaneous tumor, and knockdown of LINC00460 inhibited the tumor growth (Fig. 4B-D). Next, MC38 cells with Luc-vector and Luc-LINC00460 were used to construct CRC liver metastases in 8-week-old male C57BL/6J mice. After 20 days, bioluminescence imaging showed that the Luc-LINC00460 group had higher fluorescence intensity than the Luc-vector group (Fig. 4E). Then the mice were sacrificed and the liver tissues of Luc-LINC00460 were significantly enlarged and had more metastatic lesions than Luc-vector group (Fig. 4F-G).

Additionally, MC38 cells with Luc-sh-NC and Luc-sh-LINC00460 were used to construct CRC lung metastases in 8-week-old male C57BL/6J mice. On day 16, two mice were found dead, the others were used for bioluminescence imaging and the results showed that the Luc-sh-LINC00460 group had less fluorescence intensity than Luc-sh-NC (Fig. 4H). There was a slight reduction in volume in Lus-sh-Linc00460 mice, with no significant difference in weight (Fig. 4I-J).

Moreover, hematoxylin and eosin (H&E) staining showed that upregulation of LINC00460 increased the angiogenesis and the number of metastatic nodules (Fig. 4K). And IHC of Ki67, CD34 and CD31 showed that overexpression or knockdown of LINC00460 significantly increased or decreased the cell proliferation and angiogenesis in subcutaneous tumor, liver and lung metastases (Fig. 4K-N). Collectively, these results confirmed that LINC00460 significantly enhanced CRC tumorigenesis, metastasis and angiogenesis in vivo.

### LINC00460 facilitates the remodeling of suppressive tumor microenvironment and immune escape

Interestingly, we found that LINC00460 was positively correlated with the risk score of the stromal and immune score prognostic model of our published work [39], suggesting that LINC00460 may affect the CRC immune microenvironment (Fig. S2). Therefore, to investigate this, CRC patients in the TCGA database were divided into high- and low-LINC00460 expression groups, and four algorithms (ESTIMATE, CIBERSORT, XCELL and MCPcounter) were applied to measure the distribution ratios of different stromal and immune cell subsets. The results revealed that the high-LINC00460 group enriched more infiltrations of M2 macrophages, neutrophils, CD4 + memory T cells, endothelial cells and fibroblasts, etc. than the low-LINC00460 group (Fig. S3).

**Fig. 5:**
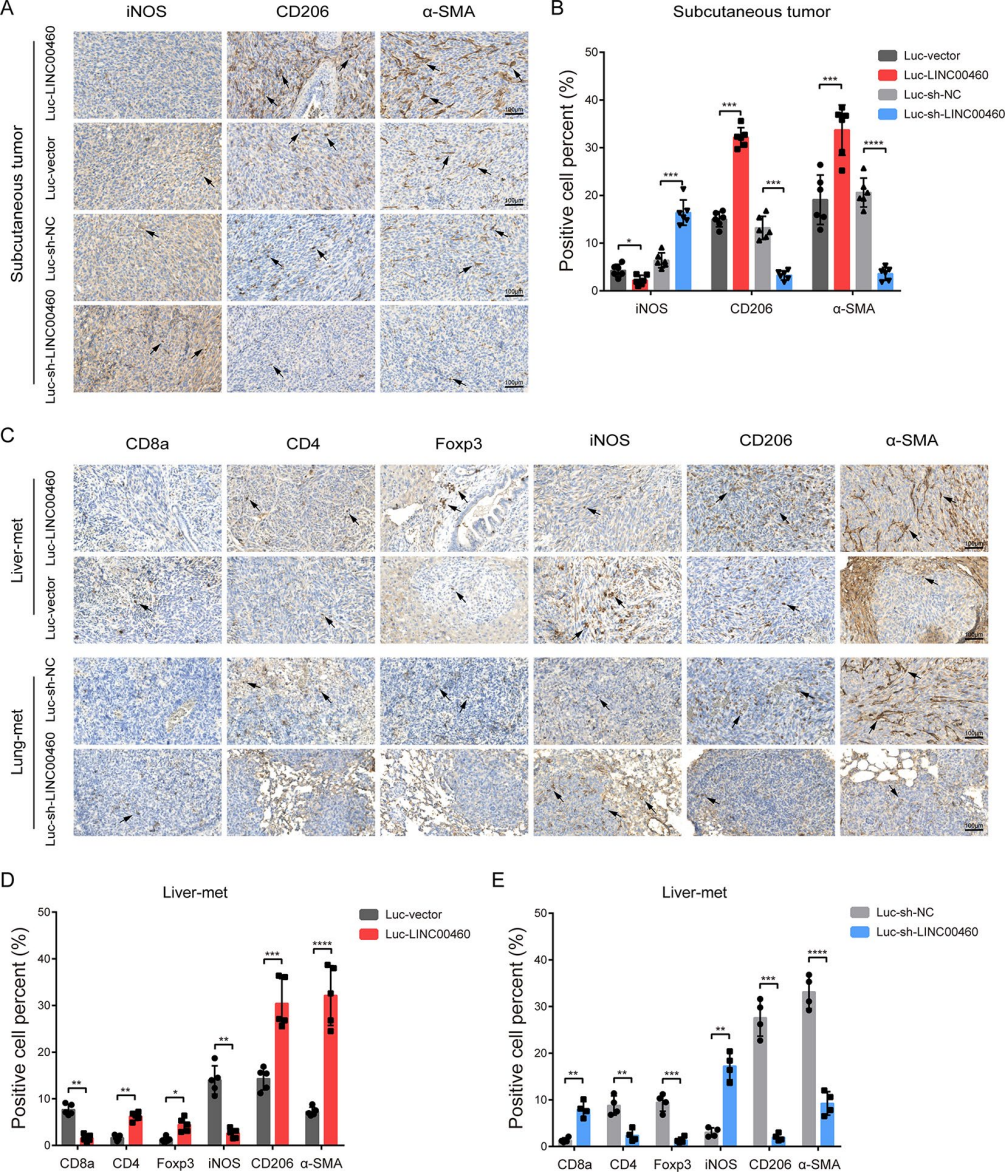
LINC00460 facilitates suppressive tumor immune microenvironment. **(A**,** B)** IHC staining of iNOS, CD206 and α-SMA in subcutaneous tumors of each group (*N* = 6). **(C**,** D**,** E)** IHC staining of CD8a, CD4, Foxp3, iNOS, CD206 and α-SMA in liver (*N* = 5) or lung (*N* = 4) metastases of each experimental group. CD8a, CD4, Foxp3, iNOS, CD206 and α-SMA were used as specific markers for CD8^+^ T cell, CD4^+^ T cell, Treg, M1 macrophage, M2 macrophage and cancer-associated fibroblast (CAF), respectively. Magnification, × 100, scale, 100 μm. Data were presented as mean ± standard deviation (SD), ns *p* ≥ 0.05, *p < 0.05, **p < 0.01, ***p < 0.001, ****p < 0.0001

Further, IHC assays were also performed on subcutaneous tumors and liver and lung metastases to depict the landscape of tumor immune microenvironment. As expected, overexpression of LINC00460 significantly hindered the infiltrations of CD8^+^ T cells and M1 macrophages, and remarkably increased the invasions of Treg, M2 macrophages and CAFs (Fig. 5A-E). Depletion of LINC00460 obtained the opposite effect (Fig. 5A-E).

Thus, these results suggest that overexpression of LINC00460 facilitates the remodeling of the suppressive tumor immune microenvironment and enhances the immune escape of CRC cells. And LINC00460 promotes CRC tumor proliferation and metastasis through immune escape. Next, we investigated the specific molecular mechanism by which LINC00460 promotes immune escape in CRC.

### LINC00460 functions as a molecular sponge for miR-186-3p

**Fig. 6:**
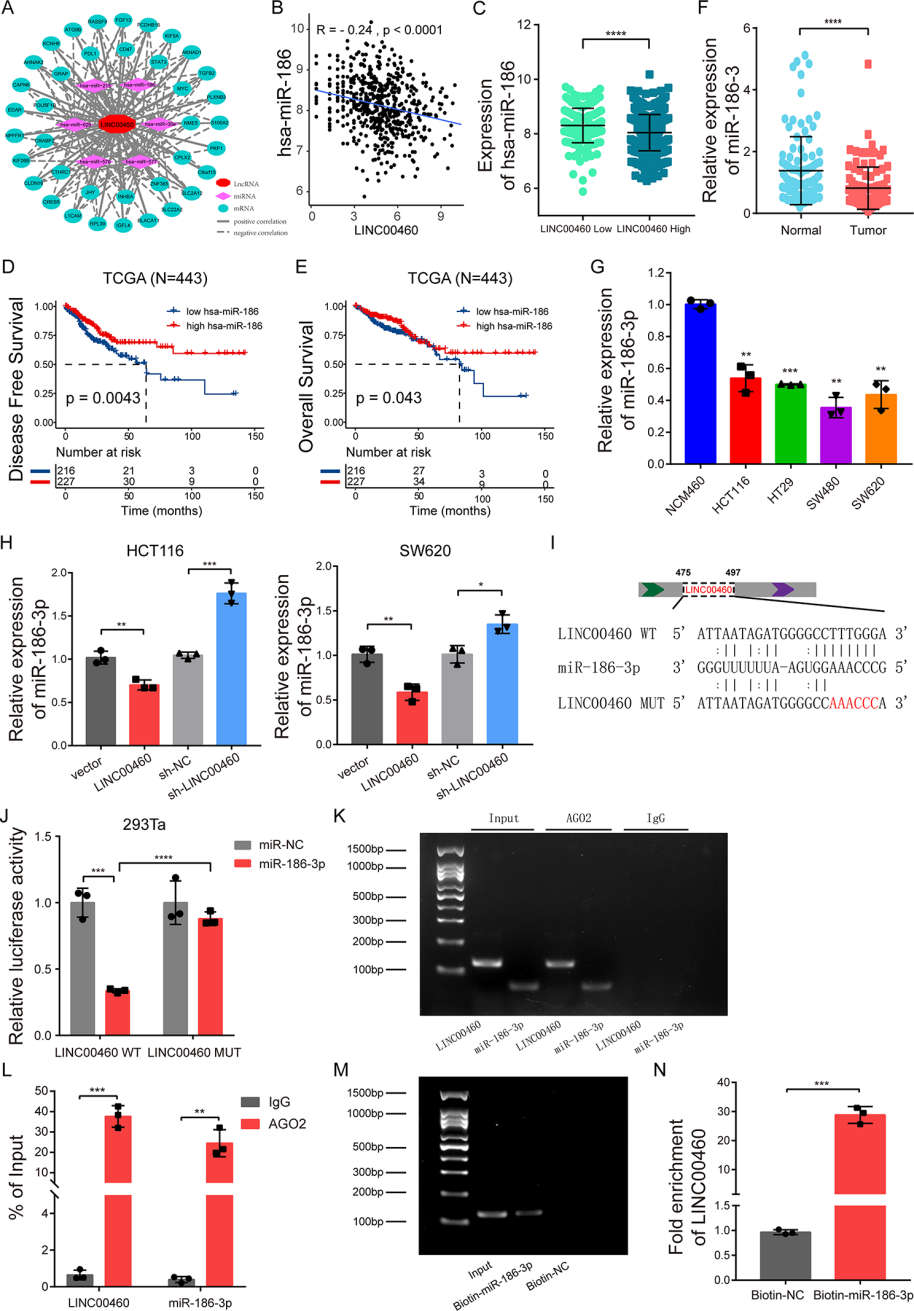
LINC00460 functions as a ceRNA for miR-186-3p. **(A**,** B)** Pearson correlation analysis between LINC00460 and has-miR-186 in TCGA database. **(C)** The expression levels of miR-186-3p classified by LINC00460 low- and high-expression group. **(D**,** E)** Kaplan-Meier analysis of the correlation between has-miR-186 and disease-free survival and overall survival in TCGA database (*N* = 443). **(F**,** G)** qRT-PCR analysis measured the expression level of miR-186-3p in 102 pairs of human CRC tumors and normal colon controls, and NCM460, HCT116, HT29, SW480 and SW620 cells (*N* = 3). **(H)** MiR-186-3p was detected by qRT-PCR after up- or down-regulation of LINC00460 in HCT116 and SW480 cells (*N* = 3). **(I)** Schematic illustration of LINC00460-WT and -MUT dual luciferase reporter vectors. **(J)** The relative luciferase activities were detected in 293Ta cells transfected with LINC00460-WT or -MUT and miR-186-3p mimic or mimic-NC, respectively (*N* = 3). **(K, L)** Anti-AGO2 RIP assay was performed in HCT116 cells, followed by nucleic acid electrophoresis and qRT-PCR to detect the enrichment ability of AGO2 on LINC00460 and miR-186-3p compared with IgG (*N* = 3). **(M, N)** RNA pull-down assay was executed in HCT116 cells, then nucleic acid electrophoresis and qRT-PCR detected the enrichment ability of miR-186-3p on LINC00460 (*N* = 3). Data were presented as mean ± standard deviation (SD), ns *p* ≥ 0.05, *p < 0.05, **p < 0.01, ***p < 0.001, ****p < 0.0001

Booming studies have proclaimed that LncRNAs act as competing endogenous RNAs (ceRNA) and microRNA sponges. We predicted miR-186-3p acted as a potential target miRNA of LINC00460 via bioinformatics analysis. The expression level of miR-186-3p was found to be significantly negatively correlated with LINC00460 (Fig. 6A-C). And Kaplan-Meier survival analysis revealed that high miR-186-3p expression correlated with increased DFS and OS (Fig. 6D-E). Additionally, qRT-PCR analysis verified that the miR-186-3p expression was significantly reduced in human CRC tissues and cell lines compared to normal controls (Fig. 6F-G). Consistently, up- or down-regulated LINC00460 significantly decreased or increased the expression of miR-186-3p in HCT116 and SW620 cells (Fig. 5H).

To confirm the above predictive analysis, a dual luciferase reporter assay was performed in 293Ta cells. LINC00460-WT and LINC00460-MUT (with or without miR-186-3p binding site) were subcloned into the luciferase reporter vector psiCHECK2 (Fig. 6I), and the result proved that miR-186-3p mimics significantly reduced the luciferase activity of the WT group but not the MUT group (Fig. 6J), suggesting that LINC00460 may have a direct interaction with miR-186-3p.

Considering that Argonaute2 (AGO2) is the key component of RNA-induced silencing complex (RISC), which can mediate target mRNA degradation, destabilization or transcriptional repression [40, 41], we performed RIP assay against AGO2 in HCT116 cells. And the results of electrophoresis and qRT-PCR suggested that LINC00460 and miR-186-3p were both effectively enriched in anti-AGO2 miRNA ribonucleoprotein complexes (miRNPs) compared with IgG (Fig. 6K-L). Further, RNA pull-down assay was also performed using a specific biotin-labeled miR-186-3p probe. As expected, nucleic acid electrophoresis and qRT-PCR detected specific enrichment of LINC00460 in miR-186-3p probe group compared to the control probe (Fig. 6M-N). The above results reveal that LINC00460 directly binds miR-186-3p, thereby reducing the level of free miR-186-3p. Taken together, these data confirm that LINC00460 acts as a molecular sponge for miR-186-3p.

### LINC00460 targets mir-186-3p to promote the expressions of MYC, CD47 and PD-L1

**Fig. 7:**
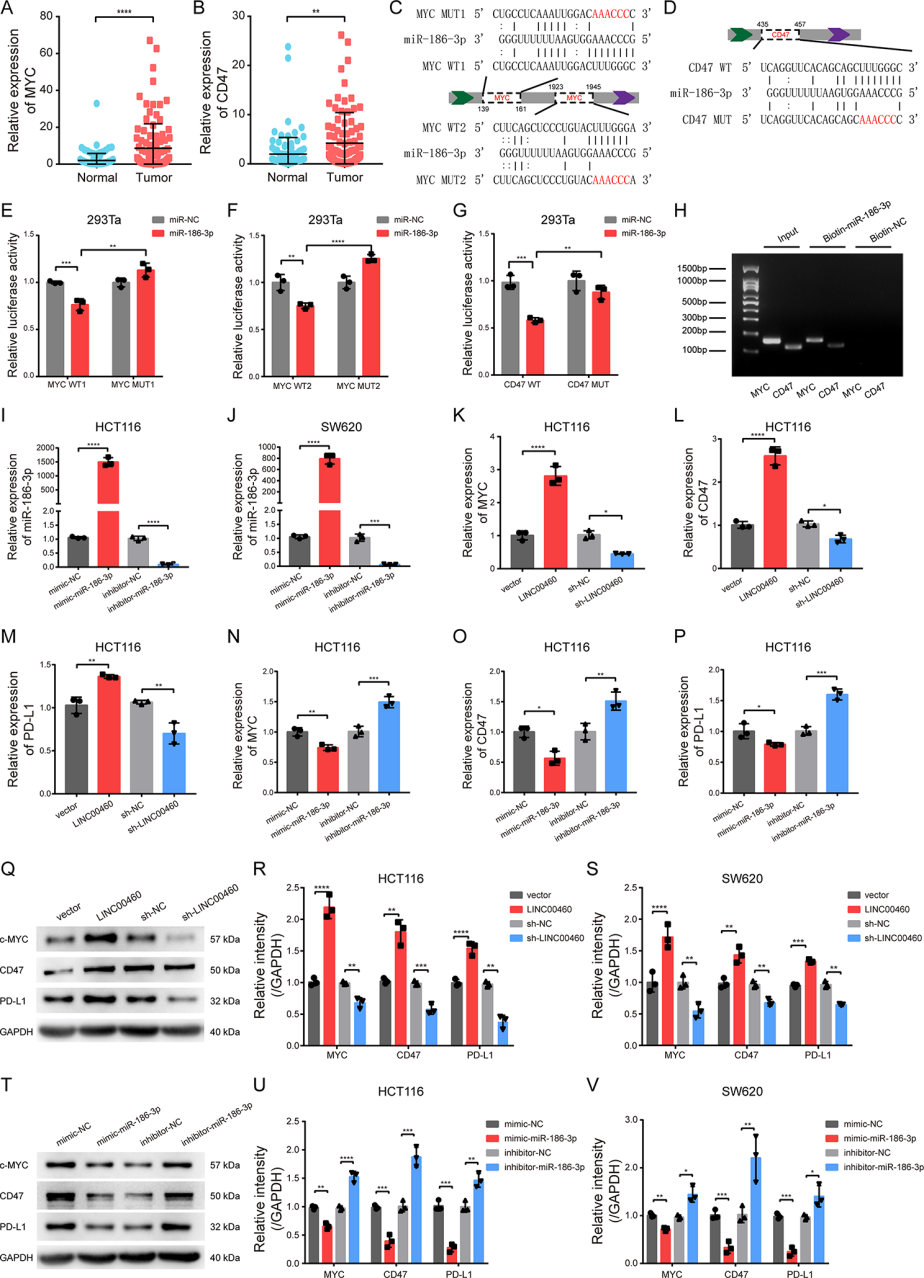
LINC00460 promotes MYC, CD47 and PD-L1 by targeting miR-186-3p in vitro. **(A, B)** The expression levels of MYC and CD47 in 102 pairs of human CRC tumors and normal colon controls were detected by qRT-PCR. **(C, D)** Schematic illustration showed the miR-186-3p binding sites with MYC and CD47 3′-UTR and the MYC/CD47-WT and -MUT dual luciferase reporter vectors. **(E, F, G)** Relative luciferase activities were detected in 293Ta cells after transfection with MYC/CD47-WT or -MUT and miR-186-3p mimic or mimic-NC, respectively (*N* = 3). **(H)** RNA pull-down assay was performed in HCT116 cells, and the enrichment ability of miR-186-3p on MYC and CD47 were measured by nucleic acid electrophoresis (*N* = 3). **(I, J)** qRT-PCR was used to detect the transfection efficiency of miR-186-3p mimics and inhibitor in HCT116 and SW480 cells (*N* = 3). **(K**,** L**,** M**,** N**,** O**,** P**,** Q**,** R**,** S**,** T**,** U**,** V)** The mRNA and protein levels of MYC, CD47 and PD-L1 in HCT116 and SW480 cells stably transfected by LINC00460 or sh-RNA and miR-186-3p mimics or inhibitor were evaluated by qRT-PCR and western bolt, respectively (*N* = 3). Data were presented as mean ± standard deviation (SD), ns *p* ≥ 0.05, *p < 0.05, **p < 0.01, ***p < 0.001, ****p < 0.0001

Intriguingly, based on TargetScan and miRBase, MYC and CD47 were found to share the same miRNA response element (MRE) of miR-186-3p with LINC00460. Data from the GEPIA2 website showed that MYC and CD47 were overexpressed in COAD and READ compared to normal tissues (Fig. S4A, D). The expression levels of MYC and CD47 were prominently increased in the high-LINC00460 group compared to the low-LINC00460 group of both TCGA and GEO databases (Fig. S4B-C, E-F). Consistently, qRT-PCR analysis of 102 pairs of human CRC and normal controls validated that the expression of MYC and CD47 was notably increased in tumors (Fig. 7A-B). Furthermore, correlation analysis of TCGA and qRT-PCR data showed that MYC and CD47 were significantly positively correlated with LINC00460 and negatively correlated with miR-186-3p (Fig. S5A-B).

To verify the binding of miR-186-3p and MYC or CD47, we subcloned two miR-186-3p binding sites in the MYC 3′-UTR and one in the CD47 3′-UTR into luciferase reporter vector psiCHECK2 (Fig. 7C-D). The results of dual luciferase reporter assays showed that miR-186-3p mimics significantly suppressed the luciferase activity of the MYC and CD47 3′-UTR WT groups, and point mutations in the MYC 3′-UTR and CD47 3′-UTR abolished the suppressive effect of miR-186-3p (Fig. 7E-G). Furthermore, RNA pull-down assays were performed and nucleic acid electrophoresis detected specific enrichments of MYC and CD47 in the miR-186-3p probe group compared to the control probe (Fig. 6H). Thus, these results indicate that MYC and CD47 are the downstream targets of miR-186-3p.

**Fig. 8:**
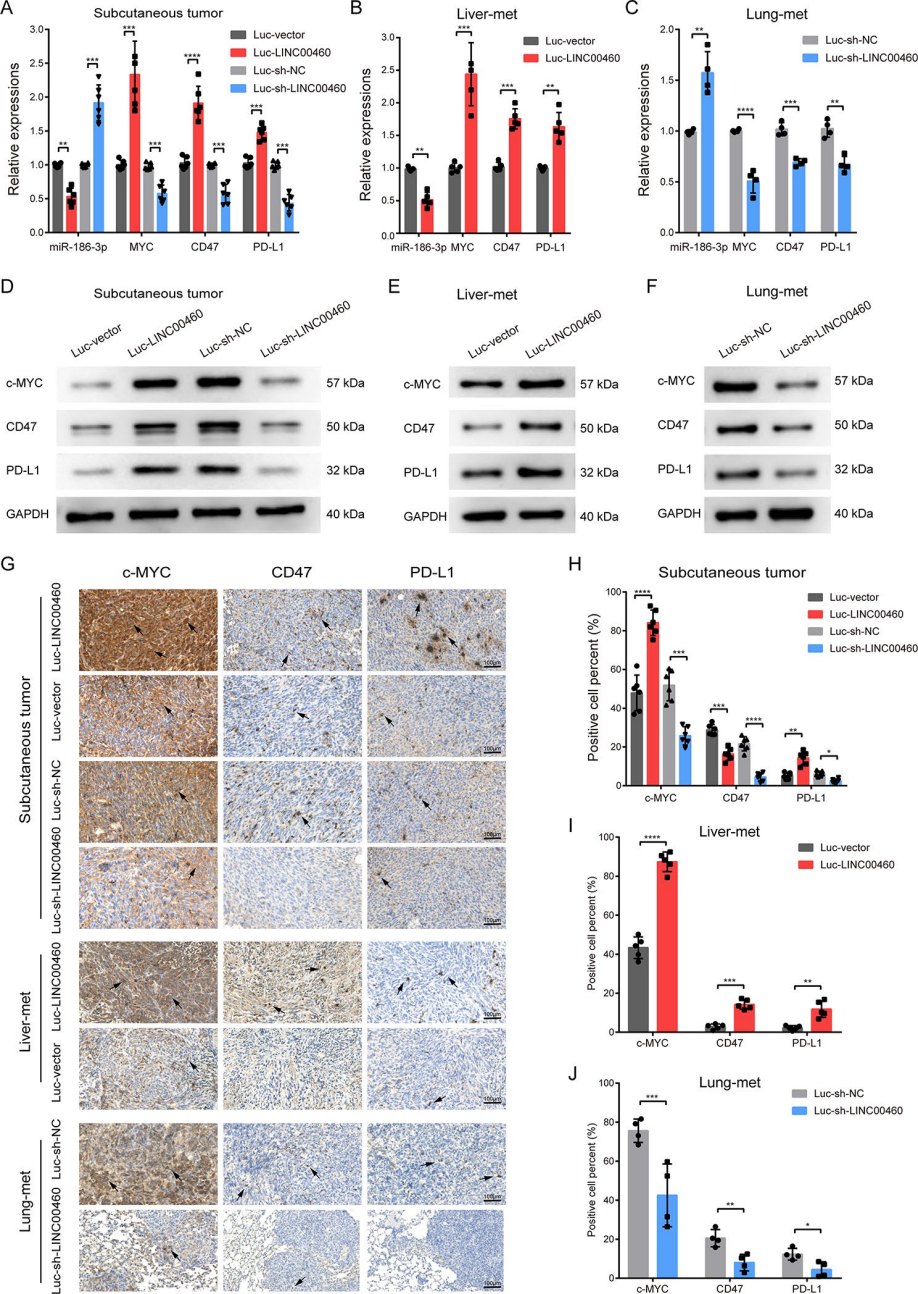
LINC00460 promotes MYC, CD47 and PD-L1 in vivo. **(A**,** B**,** C, D, E, F)** The mRNA and protein expression levels of MYC, CD47 and PD-L1 in subcutaneous tumors, liver and lung metastases of each group were detected by qRT-PCR and western bolt, respectively. **(G**,** H**,** I**,** J)** IHC assays were used to detect the ratio of MYC, CD47, PD-L1 positive cells in subcutaneous tumors, liver and lung metastatic tumors of each group. Data were presented as mean ± standard deviation (SD), ns *p* ≥ 0.05, *p < 0.05, **p < 0.01, ***p < 0.001, ****p < 0.0001

MYC is well-known to be a key transcription factor that drives the expression of many oncogenes, including the immune checkpoint molecules CD47 and PD-L1 [42–44]. MYC, CD47 and PD-L1 are considered to generate an immunosuppressive “cold” tumor microenvironment in CRC [45–47], providing insight into why LINC00460 induces immune escape and the shaping of suppressive tumor immune microenvironment. To confirm this, functional experiments were performed in vitro and in vivo. First, we overexpressed and knocked down LINC00460 in HCT116 and SW620 cells (Fig. 3B-C). Similarly, we transfected the mimic or inhibitor of miR-186-3p in HCT116 and SW480 cells, respectively (Fig. 7I-J). In both cell lines, qRT-PCR and WB assays revealed that up- or down-regulated LINC00460 notably increased or reduced the mRNA and protein levels of MYC, CD47 and PD-L1, respectively (Fig. 7K-M, Q-S, Fig. S6A-C). Conversely, miR-186-3p mimics prominently decreased the mRNA and protein levels of MYC, CD47 and PD-L1, and miR-186-3p inhibitors significantly increased the mRNA and protein levels of MYC, CD47 and PD-L1 (Fig. 7N-P, T-V, Fig. S6D-F). Consistently, the in vivo experiments based on the subcutaneous tumors, liver and lung metastases of mice, including qRT-PCR, WB and IHC, showed the same results (Fig. 8A-J).

In summary, these results confirm that LINC00460 serves as a molecular sponge for miR-186-3p to facilitate the derepression of MYC and CD47 at the post-transcriptional level, thereby promoting the expressions of MYC, CD47 and PD-L1 in CRC cells. Therefore, we revealed that LINC00460 promoted CRC cell immune escape by increasing the expressions of MYC, CD47 and PD-L1, thus enhancing the proliferation and metastasis of CRC tumors.

### LINC00460 facilitates CRC proliferation and metastasis via miR-186-3p/MYC/CD47&PD-L1 axis

**Fig. 9:**
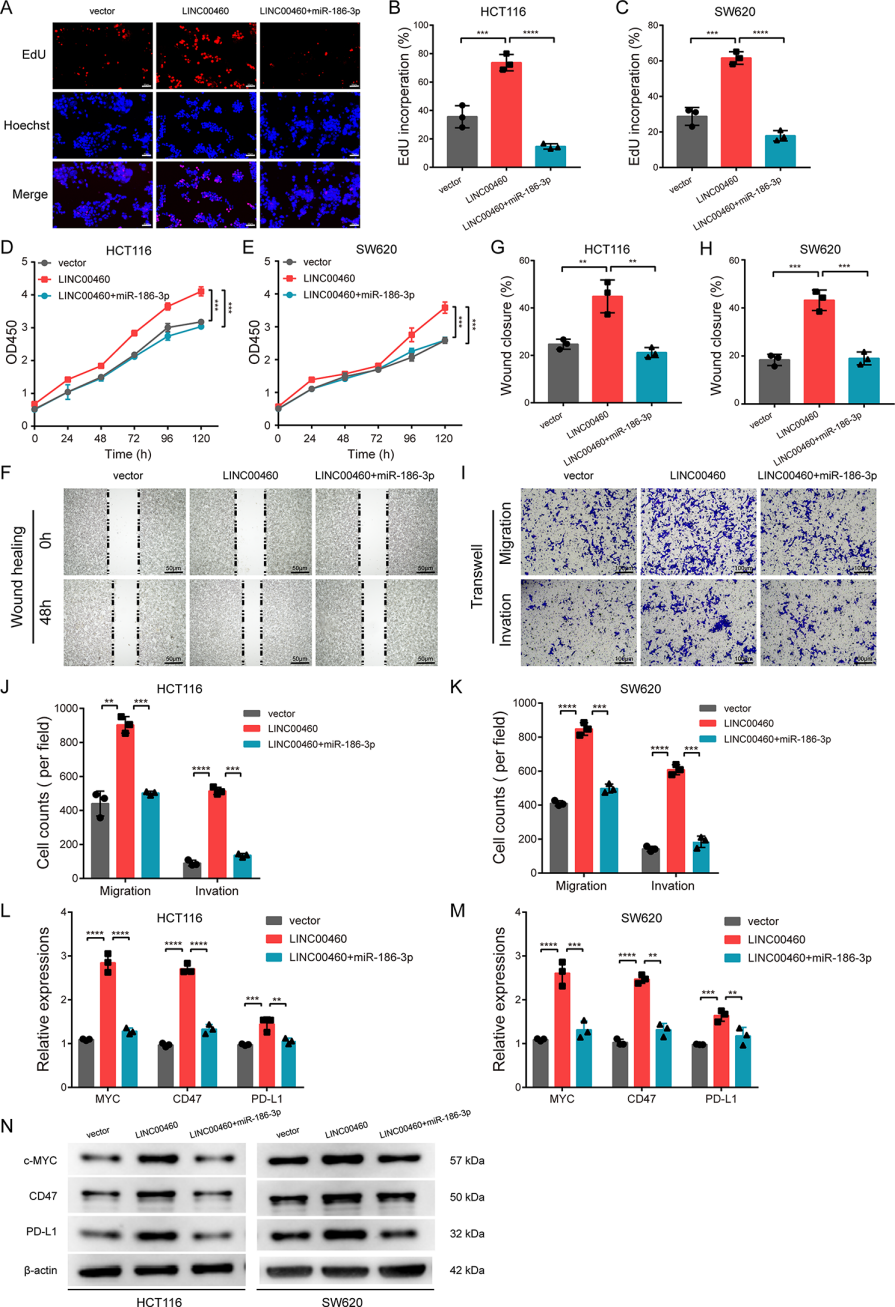
LINC00460 promotes the proliferation and metastasis of CRC through LINC00460/ miR-186-3p/MYC/CD47 axis. **(A**,** B**,** C**,** D**,** E)** EdU and CCK-8 assays were used to detect the cell proliferation of HCT116 and SW480 cells after transfection with overexpression LINC00460 vectors and miR-186-3p mimics (*N* = 3, magnification, 200 x, scale bar, 50 μm). **(F**,** G**,** H**,** I**,** J**,** K)** The cell migration and invasion were determined in transfected HCT116 and SW480 cells of each group by wound healing and transwell assays, respectively (*N* = 3, magnification, 100 x, scale bar, 100 μm). **(L**,** M**,** N)** The mRNA and protein levels of MYC, CD47 and PD-L1 were assessed by qRT-PCR and western bolt in transfected HCT116 and SW480 cells of each group, respectively (*N* = 3). Data were presented as mean ± standard deviation (SD), ns *p* ≥ 0.05, *p < 0.05, **p < 0.01, ***p < 0.001, ****p < 0.0001

To further investigate whether LINC00460 functions through the miR-186-3p/MYC/CD47&PD-L1 axis, rescue experiments were designed using miR-186-3p mimics. The results of EdU, CCK8, wound healing and transwell assays suggested that miR-186-3p mimics abolished the promoting effects of proliferation, migration and invasion of upregulated LINC00460 in HCT116 and SW620 cells (Fig. 9A-K). Moreover, qRT-PCR and WB analyses showed that overexpression of LINC00460 increased the mRNA and protein levels of MYC, CD47 and PD-L1, which could be reversed by miR-186-3p mimics (Fig. 9L-N). Taken together, these data demonstrate that LINC00460 promotes CRC proliferation and metastasis through the miR-186-3p/MYC/CD47&PD-L1 axis.

### MYC promotes LINC00460 in positive feedback loop

**Fig. 10:**
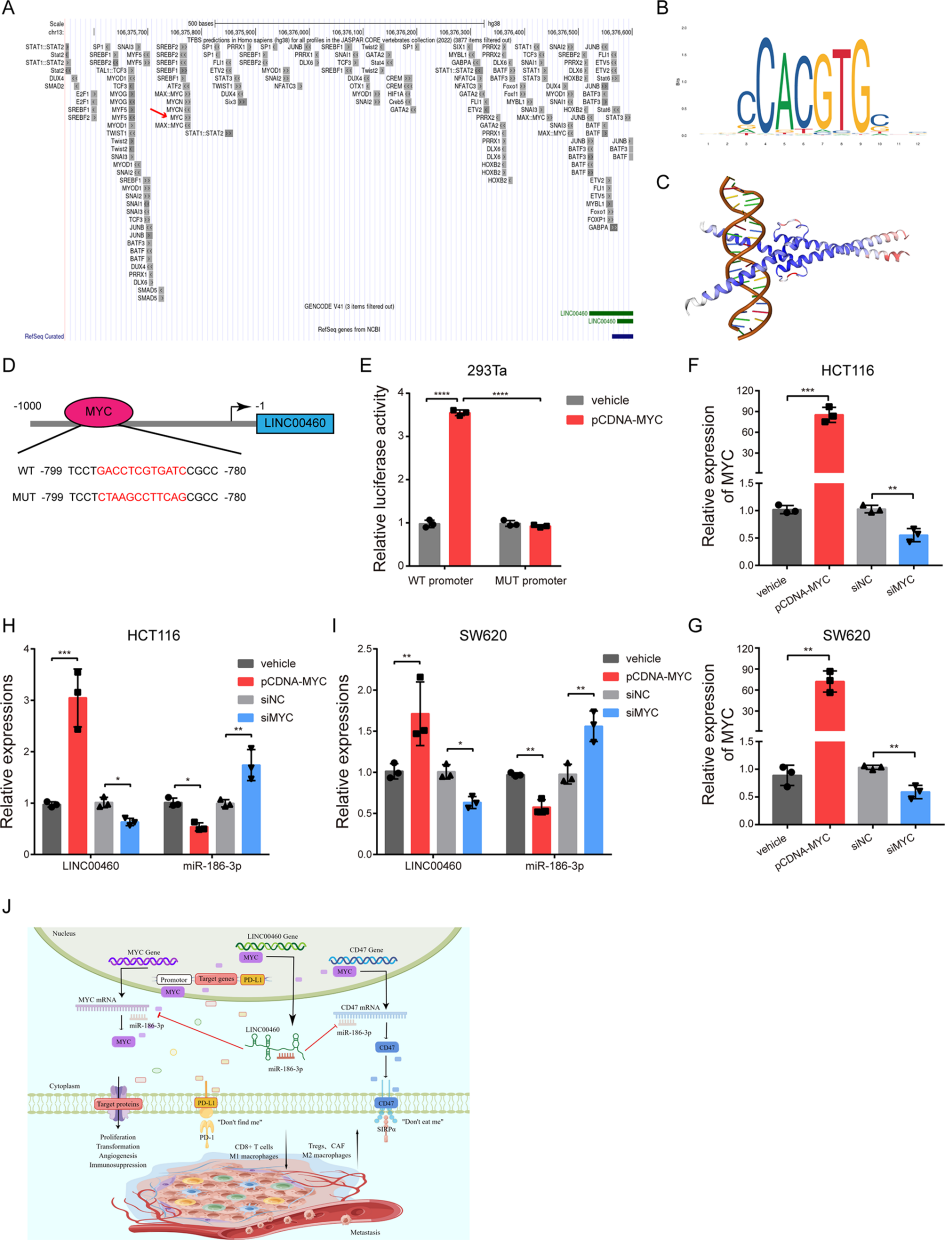
MYC upregulates LINC00460 on transcriptional level. **(A)** The UCSC database showed the potential transcription factors in 1 kb upstream sequence of the LINC00460 transcription initiation. **(B)** The JASPAR database showed the motif structure of MYC. **(C)** The Swiss Model database showed the 3D structure illustration of the binding of MYC protein and LINC00460 promoter. **(D**,** E)** Schematic illustration of the MYC binding site in the upstream sequence of the LINC00460 promoter, and relative luciferase activities were detected in 293Ta cells after transfection with WT or MUT LINC00460 promoter and pCDNA-MYC, respectively (*N* = 3). **(F**,** G)** qRT-PCR analysis measured the expression of MYC in HCT116 and SW480 cells transfected with MYC overexpression or siRNA plasmid and their control vectors. **(H, I)** The expression levels of LINC00460 and miR-186-3p were evaluated by qRT-PCR after MYC overexpression or knockdown in HCT116 and SW480 cells, respectively (*N* = 3). **(J)** The Schematic model for the major molecular mechanisms of “MYC/LINC00460/ miR-186-3p/MYC/CD47” positive feedback loop in CRC. The diagram was depicted by Figdraw website (www.figdraw.com). Data were presented as mean ± standard deviation (SD), ns *p* ≥ 0.05, *p < 0.05, **p < 0.01, ***p < 0.001, ****p < 0.0001

Based on the UCSC database, we found a potential transcription factor binding site (TFBS) for MYC in 1 kb upstream of the LINC00460 transcription initiation sequence (Fig. 10A). The motif structure of MYC and the predicted binding sequence for MYC to the promoter of LINC00460 (12 bp) were found in the JASPAR database (Fig. 10B, D). The Swiss Model database showed the 3D structure image of MYC protein binding to the TFBS of LINC00460 promoter (Fig. 10C). To verify this, a dual luciferase reporter assay was performed and showed that MYC bound to the LINC00460 promoter (Fig. 10D-E). Subsequently, functional experiments were established using the overexpression vector and siRNA of MYC (Fig. 10F-G). And qRT-PCR analysis showed that up- or down-regulation of MYC significantly enhanced or reduced LINC00460 expression in HCT116 and SW620 cells, respectively (Fig. 10H-I). Conversely, up- or down-regulation of MYC decreased or increased the expression of miR-186-3p, respectively (Fig. 10H-I).

Consequently, these observations confirmed that MYC up-regulates LINC00460 at the transcriptional level, thereby achieving a positive feedback loop: MYC/LINC00460/ miR-186-3p /MYC (Fig. 10J).

## Discussion

Over the past decades, an increasing number of articles have reported that LncRNAs play an important role in the development and progression of CRC via epigenetic, transcriptional and post-transcriptional regulation. Although it is a novel point that LncRNAs are associated with immunity, few studies have explored the detailed and in-depth molecular mechanism of LncRNAs affecting tumor immunity in CRC.

In this study, we screened the LncRNA LINC00460 by differential analysis and survival analysis. LINC00460 was first reported by Lian et al. in 2018 to affect the proliferation and apoptosis of CRC cells by inhibiting KLF2 transcription via binding to an enhancer of EZH2 and acting as a ceRNA for miR-149-5p and unblocking its inhibition of CUL4A [48]. Later, Meng et al. revealed the mechanism of LINC00460-miR-149-5p/miR-150-5p- mutated p53 feedback loop in CRC oxaliplatin resistance, providing potential therapeutic targets for CRC chemoresistance [49]. Hou et al. also found that LINC00460 promoted the proliferation and metastasis of CRC by enhancing the stability of HMGA1 mRNA through interaction with IGF2BP2 and DHX9 [50]. However, current studies haven’t revealed the effect and mechanism of LINC00460 on the tumor immune microenvironment of CRC.

Interestingly, we found that the expression level of LINC00460 was significantly positively correlated with the risk score of the stromal and immune cell-based prognostic model, suggesting that LINC00460 may serve as an immune regulator of CRC tumors. Next, we have constructively demonstrated that LINC00460 facilitated the shaping of the suppressive immune microenvironment and promoted the immune escape of CRC cells by blocking the invasion of CD8 ^+^ T cells and M1 macrophages and promoting the infiltration of Tregs, M2 macrophages and CAFs.

To explore the mechanism of LINC00460 affecting CRC immune microenvironment, we verified that LINC00460 acted as a molecular sponge for miR-186-3p, and confirmed that their downstream functional targets were MYC, CD47 and PD-L1 according to the in vitro and in vivo experiments.

MYC is a gene superfamily, whose products are mainly C-MYC with collateral N-MYC and L-MYC [51, 52], and considered to be a great coordinator of cancer growth and immune escape [45]. MYC primarily functions as a transcription factor, regulating the expression of thousands of oncogenes, which regulate the proliferation, metabolism, invasiveness, angiogenesis, autophagy, and protein and ribosome biosynthesis of CRC [53–58]. In this study, we found that MYC acted as a transcription factor of LINC00460 and upregulated the expression level of LINC00460, leading to a vicious cycle and adverse outcome of CRC patients.

Moreover, MYC also enables cancer cells to evade and suppress immune surveillance to protect their survival through the following several different mechanisms [59–61]. MYC regulates the production of several immune ligands, receptors and effector molecules, such as PD-L1, CD47, MHC classes I and II, and NKG2D [42–44, 62–64]. MYC also promotes the expression of multiple cytokines, such as CCL2, IL-13, IL-23, and CCL9, which regulate the transformation of anti-tumor M1 macrophages into pro-tumor M2 macrophages, block the activation and recruitment of B cells, natural killer (NK) cells, and CD8 ^+^ T cells, and activate mast cells and induce angiogenesis [60, 61, 65, 66].

CD47 and PD-L1 are widely studied immune checkpoint molecules that play a decisive role in the formation and maintenance of an inhibitory immune microenvironment in cancers. CD47 binds to the SIRRP-α receptor on the surface of phagocytes and transmits the “Don’t eat me” signal of innate immune response, thereby inhibiting cancer cell clearance [46, 67–69]. PD-L1 binds to the PD-1 receptor on the surface of T cells and transmits the “Don’t find me” signal of the adaptive immune response, resulting in inhibition of cancer cell killing [47, 70].

Therefore, the overexpression of these immune checkpoint molecules promoted by LINC00460 inhibited the recognition and killing of CRC cells by macrophages and cytotoxic T cells, thereby achieving immune escape of CRC cells. In addition, MYC induced the remodeling of the suppressive immune microenvironment by promoting the expression of multiple cytokines, which also promoted CRC cell immune escape.

In summary, this study clearly elucidates that LINC00460 promotes CRC immune escape through the LINC00460/miR-186-3p/MYC feedback loop, thereby enhancing the initiation and progression of CRC tumors. Targeted inhibition of LINC00460 may reduce the abundance of downstream MYC, CD47 and PD-L1 molecules, thereby inhibiting CRC tumor development and improving patient survival. Targeting LINC00460 in combination with immune checkpoint inhibitors may enhance the efficacy of CRC immunotherapy, which needs to be further verified.

There are also some limitations to the study. First of all, the human samples used in our study were from a single center in Guangzhou. Secondly, in terms of in vivo experiments, we constructed mice models of subcutaneous tumor and experimental metastatic tumor, which may have some differences from carcinoma in situ and spontaneous metastasis. Finally, LINC00460 may affect CRC tumor immunity through other pathways, and we have already evidenced some clues and planned to continue this study.

## Conclusions

Collectively, our study has systematically and innovatively confirmed that the LINC00460/miR-186-3p/MYC feedback loop consistently promotes CRC cell immune escape by enhancing CD47 and PD-L1 expressions, thereby promoting the proliferation and metastasis of CRC tumors. Our study provides a novel therapeutic strategy and target for CRC patients.

### Electronic supplementary material

Below is the link to the electronic supplementary material.

Supplementary Material 1

## Data Availability

The datasets analyzed in this study are available in the GEO repository, https://www.ncbi.nlm.nih.gov/geo/, and the UCSC/TCGA-Hub repository, https://xenabrowser.net/datapages/.

## Abbreviations

CRC: Colorectal cancer
LncRNAs: Long non-coding RNAs
COAD: Colon adenocarcinoma
READ: Rectum adenocarcinoma
GEO: Gene Expression Omnibus
FFPE: Formalin-fixed and paraffin-embedded
ISH: In situ hybridization
IHC: Immunohistochemistry
qRT-PCR: Quantitative real-time PCR
WB: Western blot
RIP: RNA immunoprecipitation
ANOVA: One-way analysis of variance
sd: Standard deviation
CNV: Copy number variation
DFS: Disease-free survival
OS: Overall survival
TNM: Tumor-node-metastasis
CMS: Consensus molecular subtypes
H&E: Hematoxylin and eosin
CAF: Cancer-associated fibroblast
ceRNA: Competing endogenous RNAs
AGO2: Argonaute2
RISC: RNA-induced silencing complex
miRNPs: miRNA ribonucleoprotein complexes
MRE: miRNA response element
TFBS: Transcription factor binding sites
