# Supplementary material for: LINC00460/miR-186-3p/MYC feedback loop facilitates colorectal cancer immune escape by enhancing CD47 and PD-L1 expressions
Source: J Exp Clin Cancer Res. 2024 Aug 13;43:225. doi: 10.1186/s13046-024-03145-1 (PMC11321182; doi:10.1186/s13046-024-03145-1)
Supplement: Supplementary file 1 — Supplementary Material 1 [file 13046_2024_3145_MOESM1_ESM.docx]

**Supplementary materials**

**Supplementary Materials and Methods**

**Nuclear and** **cytoplasmic RNA extraction**

Total RNA was extracted from the nuclear and cytoplasmic fractions using the PARIS™ kit (Invitrogen, Thermo Fisher Scientific, Waltham, USA). The expression levels of LINC00460 and miR-186-3p in nuclear and cytoplasm were detected by qRT-PCR with U6 and 18S rRNA as internal reference, respectively.

**Vector construction and cell transfection**

Full-length LINC00460 was inserted into the overexpression vector PCDH-CMV-MCS-EF1-copGFP-T2A-Puro (IGEbio, Guangzhou, China), with the mock (empty) vector as a control. LINC00460 shRNA sequences were cloned to the vector pLKO.1-puro (IGEbio, Guangzhou, China), and pLKO.1-puro-scramble plasmid served as a control. Lenti-pacTM HIV Lentivirus Packaging Kit (GeneCopoeia, Guangzhou, China) and 293Ta cells were used for lentivirus packaging. 36-48h after transfection, lentivirus supernatant was collected and infected HCT116, SW620 and MC38 cells with the presence of 5μg/mL polybrene (Beyotime, Shanghai, China). After infection, 4μg/mL puromycin (Beyotime, Shanghai, China) was used for screening stably transduced cells for 2 weeks.

Subsequently, firefly luciferase gene sequence was cloned into pCDNA3.1(+) vector (IGEbio, Guangzhou, China), and lentivirus packaging and transfection were consistent with the above. Lentivirus supernatant was incubated with stably MC38-vector, MC38-LINC00460, MC38-sh-LINC00460 and MC38-sh-NC cells for 24h. After infection, 0.5 mg/mL G418 (Aladdin, G301863) was used to select stably transduced Luc-vector, Luc-LINC00460, Luc-sh-LINC00460 and Luc-sh-NC in MC38 cells for 2 weeks.

The CDS region of MYC gene coding region was cloned into the overexpression vector pCDNA3.1(+) (IGEbio, Guangzhou, China), with the empty vector as a control. SiRNAs targeting the back splice junction site of MYC and siRNA-NC were synthesized by IGEbio (IGEbio, Guangzhou, China). The mimics and inhibitors of miR-186-3p (IGEbio, Guangzhou, China) were transfected into target cells by Lipofectamine 2000 (Invitrogen, Waltham, USA).

The efficiency of gene overexpression and knockdown was assessed by qRT-PCR. The sequences of shRNAs, siRNAs, and miRNA mimics and inhibitor were listed in Supplementary Table S3.

**In situ hybridization (ISH) and immunohistochemistry (IHC)**

The in-situ hybridization of LINC00460 was performed on FFPE sections followed by the manufacturer’s instructions using Dig-labeled LINC00460 probe designed and synthesized by Servicebio (Wuhan, China). The slides were scanned with Aperio CS2 (Leica, Wetzlar, Germany) at 200x magnification. The images were independently evaluated using software Halo and scored by two individuals blinded to the clinical parameters. The ISH scores were evaluated by semi-quantitative assessment described by Guo, Y. et al.^[38]^. The probe sequence was listed in Supplementary Table S2.

For IHC assays, FFPE sections were incubated with primary antibodies against Ki67 (abcam, ab16667), CD34 (abcam, ab8158), CD31 (abcam, ab282746), CD8a (CST, 60168), CD4 (CST, 25229), Foxp3 (abcam, ab215206), iNOS (abcam, ab15323), CD206 (CST, 24595), α-SMA (CST, 19245), c-MYC (abcam, ab32072), CD47 (Invitrogen, PA5-116827) and PD-L1 (abcam, ab233482) according to the manufacturer’s instructions, respectively, followed by secondary antibodies and DAB and hematoxylin staining. The slides were scanned with Aperio CS2 (Leica, Wetzlar, Germany) at 200x magnification.

**Total RNA isolation and quantitative real-time PCR (qRT-PCR)**

Total RNA was isolated using RNAiso Plus (Takara, D9108A) and the RNA purity and concentration were measured using NanoDrop One (Thermo Fisher Scientific, Waltham, USA) ultramicro ultraviolet spectrophotometer. cDNA was synthesized using the PrimeScript RT reagent kit (Takara, RR047A). Realtime PCR was carried out on LightCycler 96 Real-Time PCR system (Roche Diagnostics, Basel, Switzerland) using the SYBR Green Master Mix Kit (AG, AG11701). The relative quantification values of RNA were calculated using the 2^−ΔΔCt^ method using 18S rRNA as an internal reference. The primer sequences for qRT-PCR were provided in Supplementary Table S4.

**Cell proliferation, migration and invasion assays**

CRC Cell proliferation was detected by colony formation assays, cell counting kit-8 (APExBIO, K1018-T) and EdU imaging kits (APExBIO, K1075) according to the manufacturer's instructions. Cell migration and invasion were assessed by wound healing and trans-well assays. 2 × 10^4^ stably transfected CRC cells were seeded into the upper chamber without Matrigel for migration assays, while 1 × 10^5^ CRC cells were seeded into the upper chamber with matrigel (Corning, 356234) for invasion assays, and 500 μl DMEM complete medium was added to the bottom chamber. After 24–48 hours, cells on the compartment were fixed in 4% paraformaldehyde (Biosharp, Shanghai, China) and stained with crystal violet (Beyotime, Shanghai, China), then photographed with an optical microscope (Olympus, Tokyo, Japan) and counted by ImageJ.

**Western blot (WB)**

Total proteins were extracted by RIPA lysis buffer (Beyotime, P0013B) and quantified by BCA protein assay kit (Thermo Fisher, 23227) according to the manufacturer's instructions. Equal amounts of protein were separated on SDS-PAGE (Beyotime, P0015F) gels and then transferred to PVDF membranes (Sigma-Aldrich, Roche 03010040001). After blocked with 5% non-fat powdered milk (BBI, A600669-0250) in TBST, PVDF membranes were incubated with primary antibodies against c-MYC (abcam, ab32072), CD47 (Invitrogen, PA5-116827) and PD-L1 (abcam, ab233482) for 2 h at 37°C, and with anti-GAPDH (abcam, ab9485) or anti-β-actin (abcam, ab 8226) as an internal reference. Then the PVDF membranes were incubated with secondary antibodies for 1 h at 37°C. The intensity of the bands was analyzed using a chemiluminescence substrate kit (Thermo Fisher, 34577).

**Luciferase activity assays**

To study the binding sites of ceRNA network, LINC00460, MYC-3'UTR, CD47-3'UTR and their miR-186-3p binding sites’ wild-type or mutant versions were synthesized and inserted into the dual luciferase reporter vector psiCHECK2 (IGEbio, Guangzhou, China). The recombinant plasmids were named LINC00460-WT/MUT, MYC-WT/MUT and CD47-WT/MUT, respectively. The recombinant plasmids and miR-186-3p mimics or NC were co-transfected into 293Ta cells.

To investigate the binding of transcription factor to LINC00460 promoter, wild-type and mutant sequences of the binding site of MYC to LINC00460 promoter were synthesized and added to luciferase reporter vector PGL3-BASIC (IGEbio, Guangzhou, China). The recombinant plasmid was named pGL3-LINC00460-Promoter. 293Ta cells were co-transfected with pGL3-LINC00460-promoter, PRL-TK and pCDNA-MYC. The experiments followed the manufacturer's protocol of the dual luciferase reporter assay kit (Beyotime, RG027). Relative luciferase activities were measured with multifunctional microplate reader BioTEK-800TS (Agilent, Agilent Technologies, California, USA).

**RNA immunoprecipitation (RIP)**

According to the manufacturer's instructions of RNA-Binding Protein Immunoprecipitation Kit (BersinBioTM, Bes5101), RIP experiments were conducted with AGO2 antibody (abcam, ab186733). HCT116 cells were lysed in complete RIP lysis buffer, and the cell extract was incubated with protein A/G agarose beads conjugated with AGO2 antibody or control IgG overnight at 4 °C. Beads were washed and incubated with proteinase K to remove proteins. Finally, purified RNA was subjected to nucleic acid electrophoresis and qRT-PCR analysis.

**RNA pull-down assay**

The 3′ biotin-labeled miR-186-3p pulldown probe or NC pulldown probe were synthesized by Sangon Biotech (Guangzhou, China). HCT116 cells were lysed with RIPA (Beyotime, P0013B) and incubated with miR-186-3p probes or NC probe. Then cell lysates were incubated with streptavidin-coated magnetic beads (Invitrogen, 65305) to pull down the biotin-labelled RNA complex. The RNA was purified with RNAiso Plus (Takara, D9108A). Then the abundance of LINC00460, MYC and CD47 was assessed by nucleic acid electrophoresis and qRT-PCR analysis. The probe sequences were listed in Supplementary Table S2.

**Supplementary Figures**


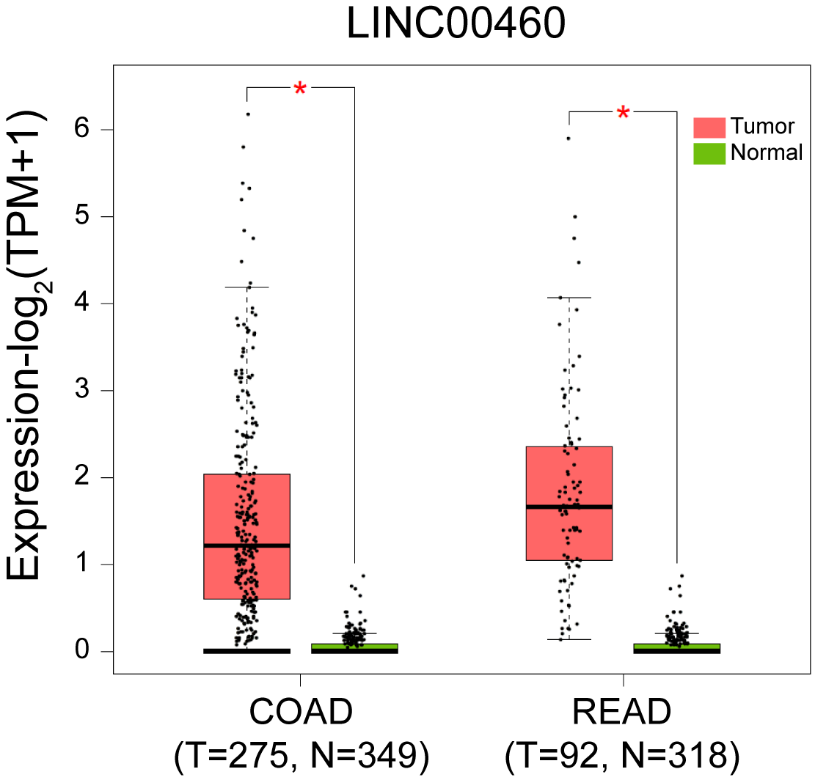


Fig.S1 The expression level of LINC00460 in COAD and READ compared to normal colon tissues in GEPIA2 website.


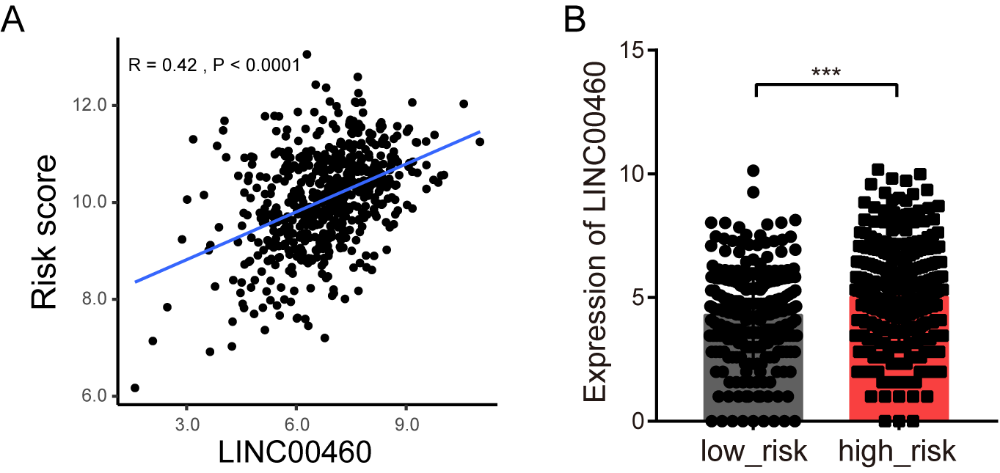


Fig.S2 The correlation of LINC00460 expression and risk score. **(A)** The scatter diagram of Pearson correlation of LINC00460 expression and risk score. **(B)** The LINC00460 expression of low- and high-risk score groups.


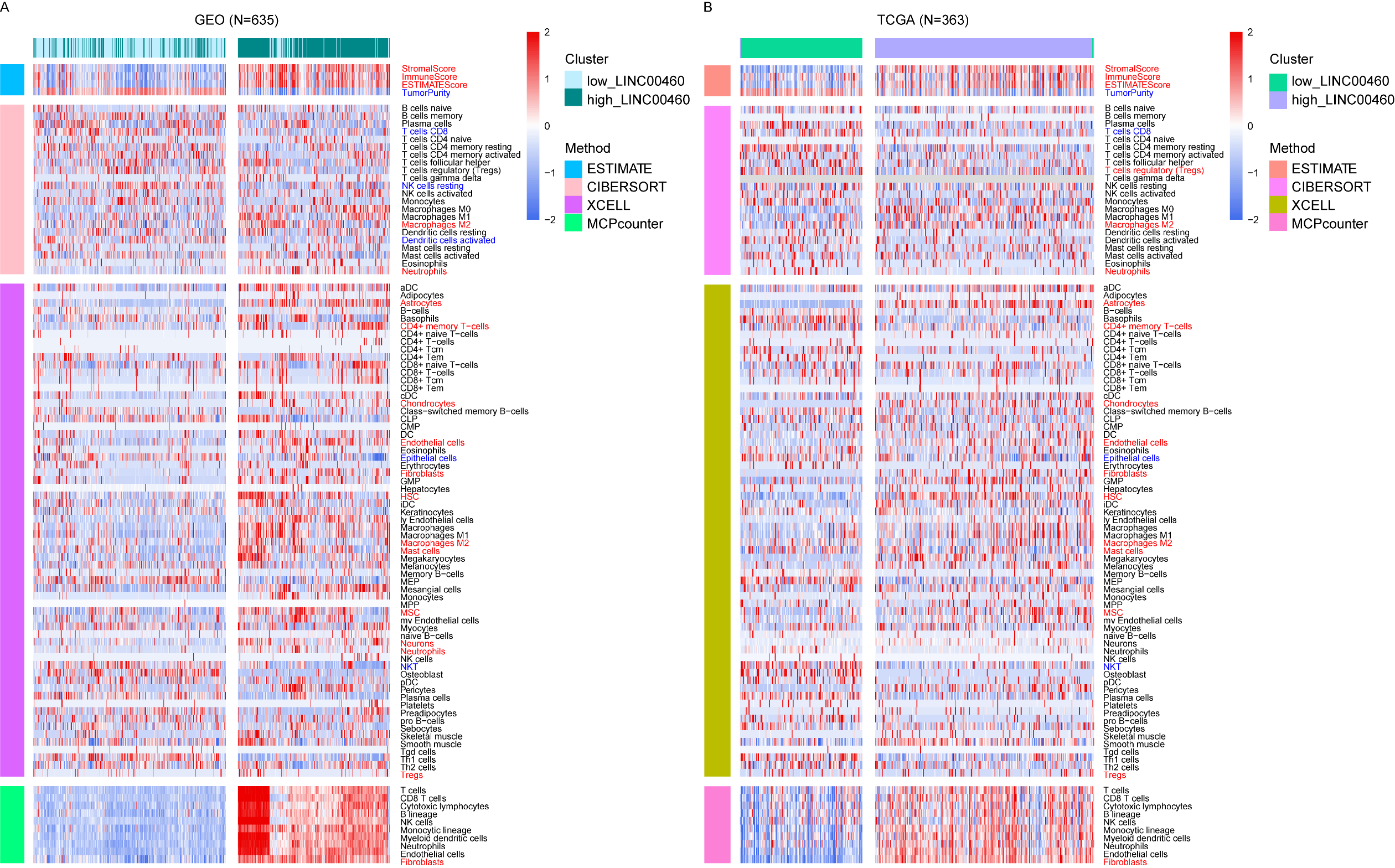


Fig.S3 The distribute ratios of various stroma and immune cell subsets were measured by four algorithms (ESTIMATE, CIBERSORT, XCELL and MCPcounter) of low- and high-LINC00460 expression groups in TCGA database.


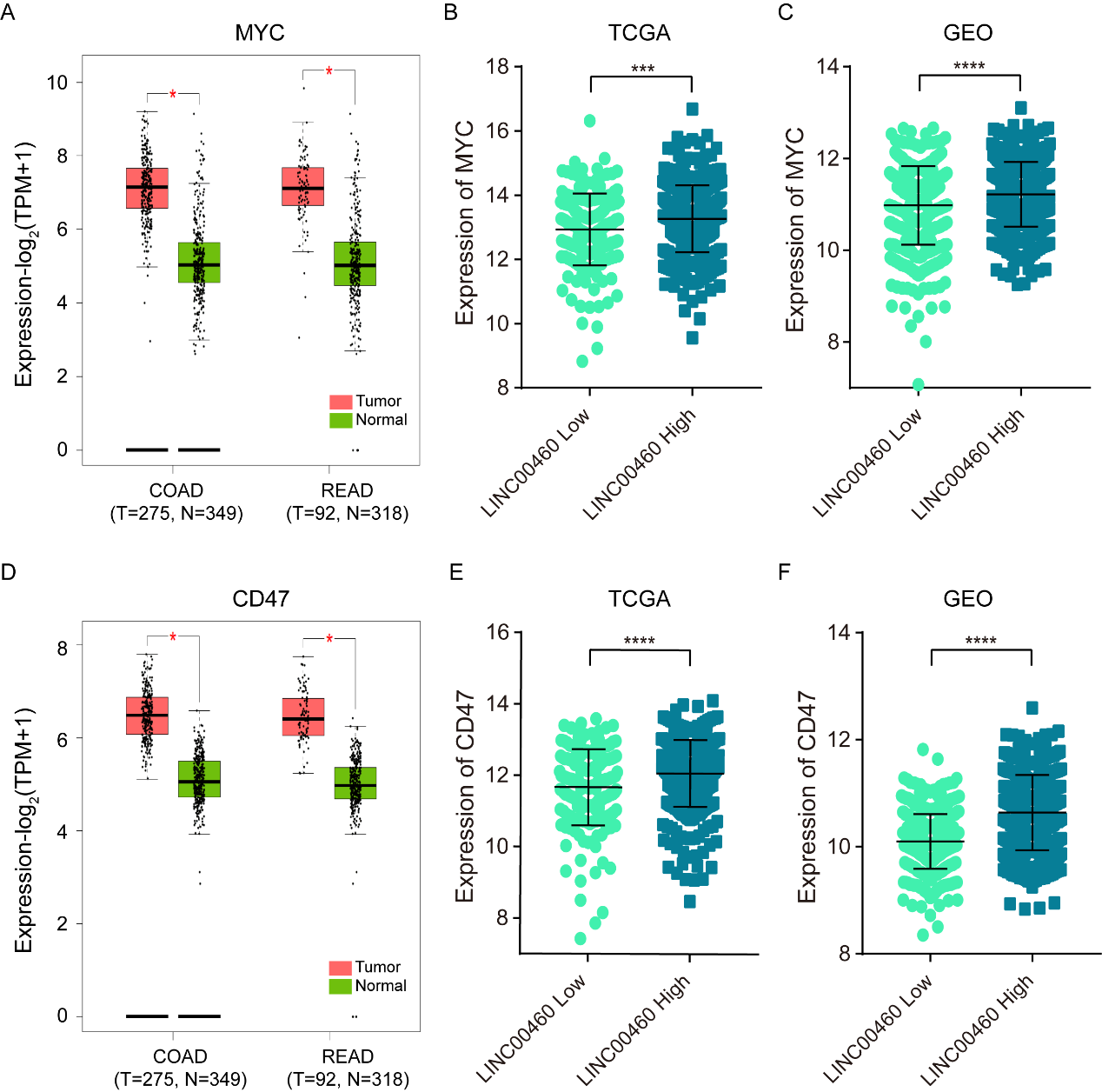


Fig.S4 The expression of MYC and CD47 in CRC and their correlation with LINC00460. **(A, D)** The expression levels of MYC and CD47 in COAD and READ compared to normal colon tissues in GEPIA2 website. **(B, C, D, E)** The expression levels of MYC and CD47 in low- and high- LINC00460 expression groups from TCGA and GEO databases.


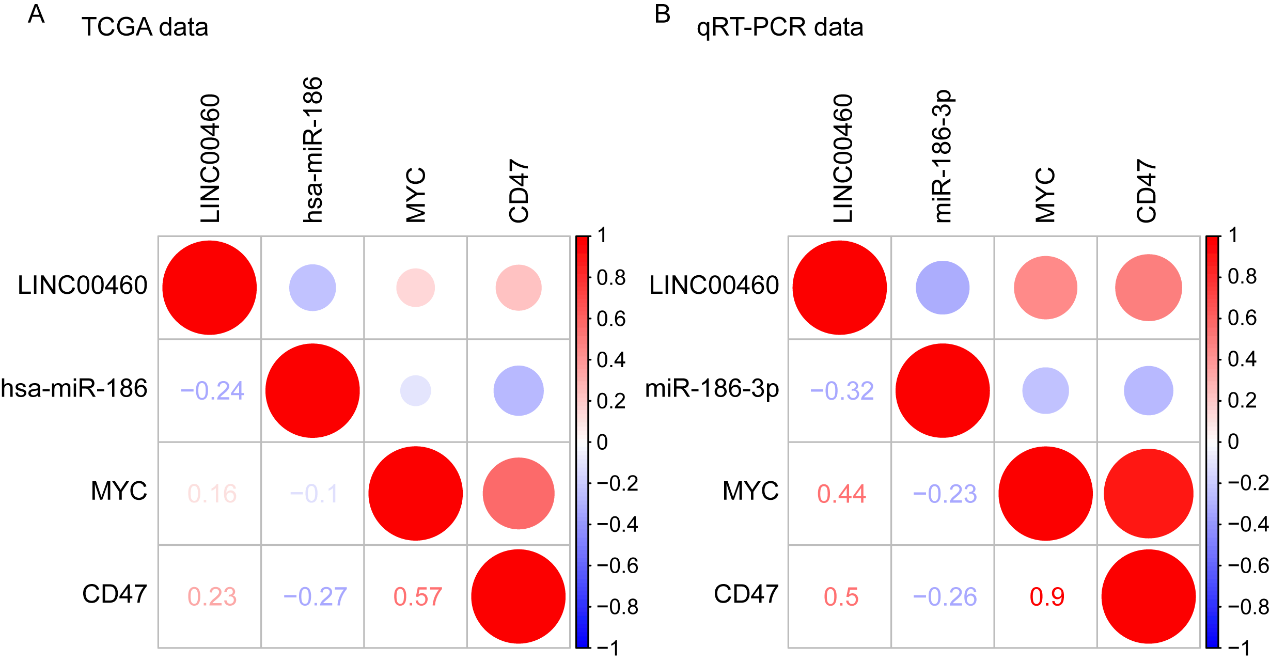


Fig.S5 The correlation of LINC00460, miR-186-3p, MYC and CD47 in CRC. **(A)** TCGA databases. **(B)** qRT-PCR analysis of human CRC tissues.


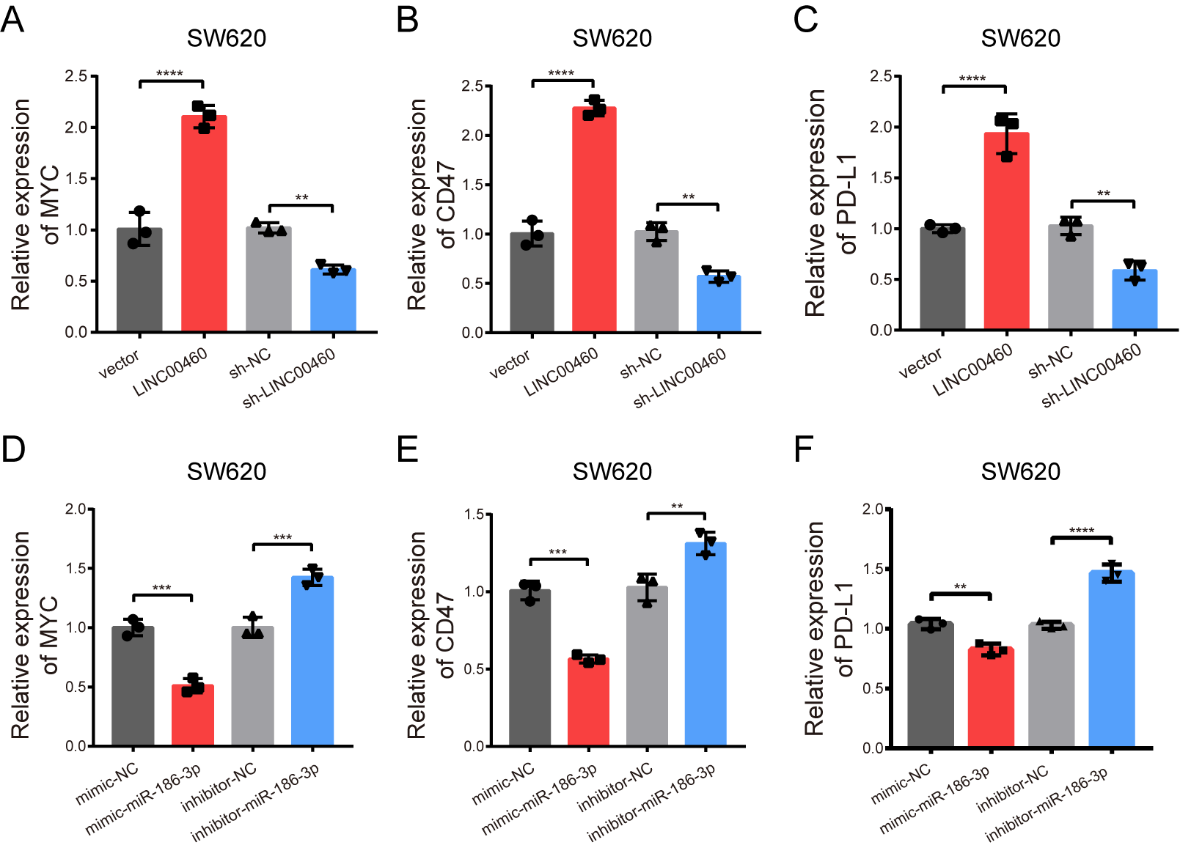


Fig.S6 The expression levels of MYC, CD47 and PD-L1 in transfected SW620 cells. **(A, B, C)** The expression levels of MYC, CD47 and PD-L1 after overexpression or knockdown of LINC00460 in SW620 cells. **(B)** The expression levels of MYC, CD47 and PD-L1 in SW620 cells transfected with miR-186-3p mimics or inhibitor.

**Supplementary Tables**

**Table S1 The data source of included samples from public databases.**

| **Database** | **Series accession** | **Sample number** | **Included samples** | **Normal** | **Tumor** | | **Metastasis** |
| --- | --- | --- | --- | --- | --- | --- | --- |
| **GEO** | GSE109454 | 12 | 12 | 6 | 6 | 0 | |
|  | GSE87211 | 363 | 363 | 160 | 203 | 0 | |
|  | GSE41568 | 133 | 119 | 0 | 39 | 80 | |
|  | GSE131418 | 1135 | 1135 | 0 | 878 | 257 | |
|  | GSE29621 | 65 | 65 | 0 | 65 | 0 | |
|  | GSE38832 | 122 | 122 | 0 | 122 | 0 | |
|  | GSE39084 | 70 | 70 | 0 | 70 | 0 | |
|  | GSE39582 | 585 | 585 | 19 | 566 | 0 | |
|  | GSE72970 | 124 | 124 | 0 | 124 | 0 | |
| **UCSC Xena** | GDC TCGA COAD | 512 | 469 | 39 | 430 | 0 | |
|  | GDC TCGA READ | 177 | 163 | 9 | 154 | 0 | |
| **Total** | - | **3298** | **3227** | **233** | **2657** | **337** | |

**Table S2. The sequences of ISH, FISH and pulldown probes used in this study.**

| **Gene name** | **Probe (5’-3’)** |
| --- | --- |
| LINC00460 (ISH) | DIG-GGAGATGAGTCCCCCTGGCTGAGGCATTTCTAACA-DIG |
| miR-186-3p pulldown | 5’ GCCCAAAGGUGAAUUUUUUGGG- Biotin 3’ |
| NC pulldown | 5’ CCCAAAAAAUUCACCUUUGGGC- Biotin 3’ |

**Table S3 The sequence lists of shRNAs, siRNAs, miRNA mimics and inhibitor.**

| Gene name |  | Sequence (5’-3’) | modification |
| --- | --- | --- | --- |
| sh-LINC00460-1 |  | CCGGGAAAGACTGAGCGTGGGAAAGCTCGAGCTTTCCCACGCTCAGTCTTTCTTTTTGAATT |  |
| sh-LINC00460-2 |  | CCGGGCTAAGACCTAATAGCCAATACTCGAGTATTGGCTATTAGGTCTTAGCTTTTTGAATT |  |
| sh-LINC00460-3 |  | CCGGGCCATCCACTTCAAAGTATTCCTCGAGGAATACTTTGAAGTGGATGGCTTTTTGAATT |  |
| sh-LINC00460-4 |  | CCGGGCCTCTGAAATGGTGACAATACTCGAGTATTGTCACCATTTCAGAGGCTTTTTGAATT |  |
| sh-LINC00460-5 |  | CCGGGGTACCCAGACATTGTTATGACTCGAGTCATAACAATGTCTGGGTACCTTTTTGAATT |  |
| si-MYC-1 |  | CCAACAGGAACUAUGACCUCGACUA |  |
| si-NC-1 |  | CCAAGGAAUCACAGUGCUCACACUA |  |
| si-MYC-2 |  | GAGACAUGGUGAACCAGAGUUUCAU |  |
| si-NC-2 |  | GAGUAGGGUAACCGAGAUUUCACAU |  |
| si-MYC-3 |  | AACACAAACUUGAACAGCUACGGAA |  |
| si-NC-3 |  | AACAAAUUCAAGGACAUCGCACGAA |  |
| miR-186-3p mimics | sense | GCCCAAAGGUGAAUUUUUUGGG |  |
|  | antisense | CCCAAAAAAUUCACCUUUGGGC |  |
| mimics NC | sense | UCACAACCUCCUAGAAAGAGUAGA |  |
|  | antisense | UCUACUCUUUCUAGGAGGUUGUGA |  |
| miR-186-3p inhibitor |  | CCCAAAAAAUUCACCUUUGGGC | 2’Ome |
| inhibitor NC |  | UCUACUCUUUCUAGGAGGUUGUGA | 2’Ome |

**Table S4 The sequences of primers used in this study.**

| **Gene name** | **Sequence (5’-3’)** |
| --- | --- |
| LINC00460 Forward | CTTTCCCACGCAGTGGATGA |
| LINC00460 Reverse | GAATGCGTCTTCTTTCCCACG |
| miR-186-3p RT-primer | GTCGTATCCAGTGCAGGGTCCGAGGTATTCGCACTGGATACGACCCCAAA |
| miR-186-3p Forward | GCCGAGGCCCAAAGGTGAATTT |
| miR-186-3p Reverse | CAGTGCAGGGTCCGAGGTAT |
| MYC Forward | GTCAAGAGGCGAACACACAAC |
| MYC Reverse | TTGGACGGACAGGATGTATGC |
| CD47 Forward | TCCGGTGGTATGGATGAGAAA |
| CD47 Reverse | ACCAAGGCCAGTAGCATTCTT |
| PDL1 Forward | GGACAAGCAGTGACCATCAAG |
| PDL1 Reverse | CCCAGAATTACCAAGTGAGTCCT |
| 18S rRNA Forward | CAGCCACCCGAGATTGAGCA |
| 18S rRNA Reverse | TAGTAGCGACGGGCGGTGTG |
| GAPDH Forward | GGAGCGAGATCCCTCCAAAAT |
| GAPDH Reverse | GGCTGTTGTCATACTTCTCATGG |
| β-Actin Forward | CATGTACGTTGCTATCCAGGC |
| β-Actin Reverse | CTCCTTAATGTCACGCACGAT |
| U6 RT-primer | CGCTTCACGAATTTGCGTGTCAT |
| U6 Forward | GCTTCGGCAGCACATATACTAAAAT |
